# High-efficiency multi-scale holographic volumetric 3D printing with a phase light modulator

**DOI:** 10.1038/s41377-026-02331-4

**Published:** 2026-05-19

**Authors:** Maria Isabel Álvarez-Castaño, Riccardo Rizzo, Viola Sgarminato, Ye Pu, Christophe Moser

**Affiliations:** 1 Laboratory of Applied Photonics Devices, School of Engineering, Ecole Polytechnique Fédérale de Lausanne, Lausanne, CH-1015 Switzerland; 2 Present Address: BIOINSIDE Lab, Department of Mechanical and Aerospace Engineering (DIMEAS), Politecnico di Torino, Turin, 10129 Italy

**Keywords:** Displays, Lithography

## Abstract

Light-based 3D printing with photocurable resins enables the rapid fabrication of complex structures with high resolution and fidelity. Tomographic Volumetric Additive Manufacturing (TVAM) employs a digital micromirror device (DMD) to project amplitude light patterns into rotating resin volumes, producing 3D geometries through photopolymerization. Typically, the light projection efficiency in such binary amplitude modulator-based systems is below a few percent. Recent advancements introduced phase encoding in TVAM using binary amplitude modulators and the Lee Hologram method, increasing axial control and boosting light efficiency to about 10%. In this work, we present the first 3D printing platform utilizing a phase light modulator (PLM), based on an array of micro-electro-mechanical piston mirrors. Compared to amplitude encoding, phase encoding with the PLM yields a 70-fold increase in laser power efficiency. By coupling this efficient light engine with a speckle reduction method in holographic volumetric additive manufacturing (HoloVAM), we experimentally demonstrate printing 3D objects across different scales from hundreds of micrometers to centimeters and with various materials from acrylate-based resins to soft hydrogels, including cell-laden hydrogels with a concentration of 1 million cells per mL. Micro-CT revealed a $\sim 30.3\,\mu {m}$ as the smallest positive feature printed. Moreover, we introduce the use of gelatin Thiol/Norbornene as a material for printing with the Holographic VAM technique, which allows us to print large-scale objects (up to $(3\,\times 3\times 4\,{{cm}}^{3})$ within 2 minutes using only a 150 mW laser diode. The PLM opens up new avenues in volumetric AM for holographic techniques using low-cost single-mode laser diodes.

## Abstract (web summary)

Demonstration of holographic volumetric printing using time‑multiplexed holograms with laterally shifted Bessel PSFs for speckle‑reduced projection and a PLM‑based light engine enabling efficient phase‑only modulation for rapid 3D fabrication of multiscale objects with improved surface quality.

## Graphical abstract

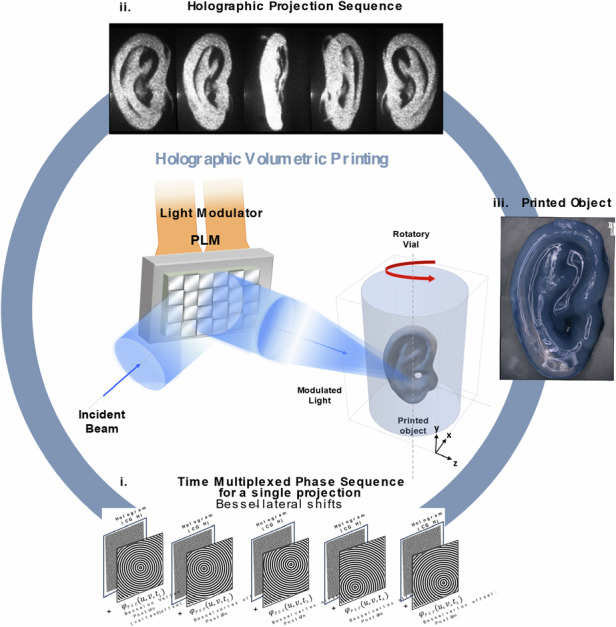

## Introduction

Three-dimensional (3D) Additive Manufacturing (AM), better known as 3D printing, has been a breakthrough in many fields such as tissue engineering^1^, regenerative medicine^2^, aerospace^3^, optical components, and many others. The first modern 3D printing method was light-based proposed by Kodama in 1981^4^ which consisted of selectively solidifying material point-by-point or layer-by layer to build three-dimensional objects. Since then, various AM methods have been developed^5–8^ using different materials. Layerless 3D technologies do not rely on layer-by-layer deposition. Such technologies are referred to as Volumetric Additive Manufacturing (VAM). Recently, several light-based VAM methods working with single–photon absorption have been developed. In reverse tomography, known as tomographic volumetric additive manufacturing (TVAM)^9,10^, an entire three–dimensional object is simultaneously solidified after sequential amplitude light patterns are displayed into a rotating photoresin vial. In Xolography^11^ and Light-Sheet 3D printing^12^, two intersecting beams of different colors are required to perform the polymerization process, while dynamic light patterns are projected into the photosensitive resin.

Currently, most VAM techniques rely on amplitude patterns, commonly displayed using Digital Micromirror Devices (DMDs). These binary amplitude light modulators operate in reflection mode by tilting their micromirrors between two states: “on” and “off”. In the “on” state, a micromirror directs light to illuminate a corresponding pixel or voxel on the printing plane, while in the “off” state light is directed elsewhere. DMDs generate grayscale patterns by rapidly toggling micromirrors between the ‘on’ and ‘off’ states, effectively controlling average light intensity. Recently, a holographic approach applied to tomographic VAM using phase encoding has been demonstrated^13^. Holographic projection enables capabilities beyond conventional amplitude-coded TVAM, including precise control of the point spread function (PSF), digital refocusing, aberration correction, and improved light efficiency. The system called HoloVAM^13^, used Lee Holograms to enable the use of the binary DMD modulator as a fast phase modulator^14,15^. However, such a phase modulator suffers from poor light efficiency ( < 10%)^13,14^.

For decades, Liquid Crystal on Silicon (LCOS) Spatial Light Modulators (SLMs) were the only commercially available option for phase spatial light modulation. This device consists of electrically addressable pixels containing long chains of liquid crystal (LC) molecules positioned between two electrodes^16,17^. The alignment of LC molecules changes in response to an applied voltage, following the direction of the electric field. This realignment alters the intrinsic birefringence, resulting in phase and thus wavefront modulation^16^. Due to the LC molecule’s viscosity, the standard frame rate range of the LCOS SLM is between 60–120 Hz. LCOS SLMs typically degrade when operated under UV light^18,19^. Recently, Texas Instruments introduced a new type of MEMS-based Phase Light Modulator (PLM) that provides phase retardation thanks to the vertical displacements of the mirrors in a piston fashion. The vertical motion of each micro-mirror can be independently addressed with a 4-bit displacement resolution (16 states or mirror levels). Currently, the evaluation module (EVM) offers frame rates of up to 1440 Hz and a fill factor of 95%, providing high speed and light efficiency. Since the PLM does not rely on LC molecules, it is less subject to pixel crosstalk, is polarization-insensitive, and stable in phase as it has no molecular relaxations^20^. Due to these advantages, PLMs are gaining significant interest in applications such as wavefront shaping, holographic projection, and augmented reality displays^21,22^.

In this work, we demonstrate the first VAM system implemented using the new MEMS phase-only modulator (i.e. PLM). We first measure and calibrate the 16 phase levels (4-bit) of the PLM using an interferometric method which uses a self-generated diffraction phase grating^23^ (see in Material and Methods section). We then measure the power efficiency of the holographic projections using the PLM and compare it with that of amplitude and phase encoding using a binary DMD. We experimentally found that pattern projections using the PLM is twice more efficient than using Lee holograms on the DMD for HoloVAM and 70-folds more efficient than amplitude. To leverage the PLM in the Holographic TVAM, we introduce a new hologram generation pipeline for computing holographic projections with reduced speckles. For each projection angle, an axicon phase pattern is used to extend the depth of field in the reconstructed intensity pattern for a larger printing range along the direction of propagation after the Fourier lens. The pattern retention achieved with the axicon phase pattern is superior to a Gaussian beam profile over the diameter of the printing vial. To reduce speckles in holographic intensity reconstruction, the reconstruction is digitally shifted laterally in a time sequence using nine distinct axicon phases, each with its vertex shifted to a different position, such that the averaging process during the printing greatly smooth out the speckles. Excellent print surface quality is achieved with a carefully chosen lateral displacement. Then to demonstrate the light efficiency, and extended capabilities of the HoloVAM light engine using a phase-only light modulator (PLM), we printed multi-scale objects with acrylate-based resins and soft hydrogels. For the first time, we showcase the capability to print large-scale objects (up to 3 cm × 3 cm × 4 cm) as well as highly concentrated cell-laden hydrogels (1 million cells/mL) by employing gelatin metacryloyl (GelMA) and gelatin thiol/norbornene with the holographic TVAM technique.

## Results

**Fig. 1:**
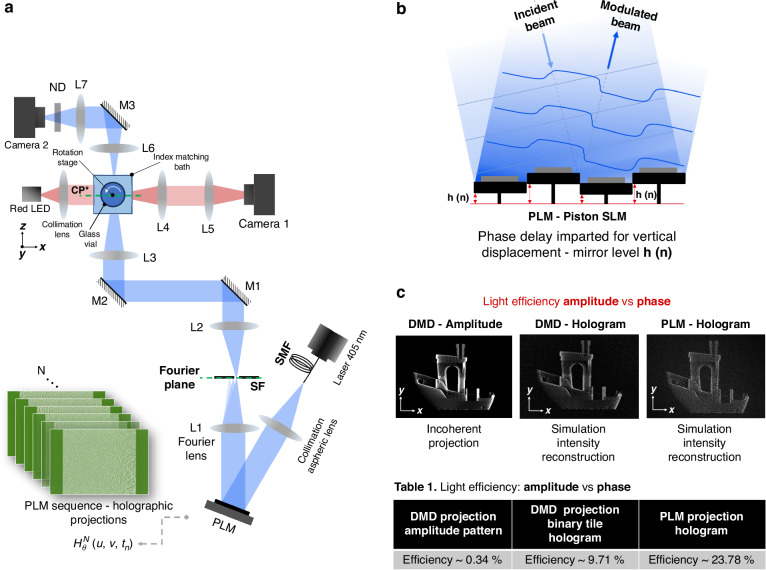
Optical configuration and light efficiency. **a** Schematic of the optical configuration for HoloVAM using a PLM as a Spatial Light Modulator. An aspherical collimation lens (CL) collimates blue light from a laser diode coupled to a single-mode fiber (SMF). After the PLM, a Fourier lens (L1) is placed, with a spatial filter (SF) at the focal plane of L1 to remove undesired diffraction orders. A 4-f system (L2 and L3) conjugates the Fourier plane (CP*), which then serves as the printing region. A glass vial (GV) acts as the sample container and a rotation stage (RS) holds the sample container. An index matching bath (IMB) is used to minimize optical distortions caused by the curved walls of the cylindrical GV. **b** Illustration of PLM row of micro-mirror pixels in piston fashion displacement to impart phase delay. **c** Light efficiency comparison

The optical setup of our holographic TVAM (HoloVAM) system using a Texas Instruments (TI) DLP67750 PLM is shown in Fig. 1a. The light source is a 405 nm single mode laser diode. The PLM is positioned in front of a Fourier lens and a glass vial containing a photosensitive resin is placed at the conjugate plane (CP) of the focal plane of the lens L1 (See the Methods section for a detailed description of the setup). During the printing process, the vial is rotated continuously at a speed determined by the PLM frame rate, and a sequence of pre-calculated full-size holograms are projected to the resin. When High-Definition Multimedia Interface (HDMI) is used, the frame rate is 720 Hz and the vial rotation speed is 30 degrees per second. When Display Port (DP) is used, the frame rate is 1440 Hz and the rotation speed is 60 degrees per second. The PLM uses 16 discrete phase levels from 0 to $2\pi$ using 4 bits of data. The phase modulation is imparted by the micromirrors’ vertical displacement as Fig. 1b illustrates. During holographic reconstruction, a linear phase ramp is added to the hologram on the PLM to separate the reconstructed intensity pattern from the zeroth order by directing the desired first diffraction order to pass through a spatial filter (SF) for printing, which blocks all other diffraction orders, including the zeroth order. Details of the PLM operation and calibration method can be found in the Methods section.

Figure 1c shows a comparison of the pattern light efficiency among two amplitude and phase-encoded projections on a DMD (Table 1. left and center columns) and phase-encoded projections using a PLM (Table 1. Right column). We construct our setup (see details in Supplementary Information Fig. S1) in such a fashion that combines a DMD and a PLM so that experimental power efficiency can be compared.

A blazed grating using a multi-level phase-only modulator between 0 and $2\pi$ has a theoretical diffraction efficiency close to 100% (98.7%) in the first order of diffraction for 16 levels. Experimentally, we achieved a diffraction efficiency for a blazed grating close to 45%. This efficiency loss is due to the fill factor of the PLM (95%), reflectivity of the mirrors at 405 nm, and a 20% light loss resulting from to the internal PLM operation, in which the micromirrors are reset to zero position for a period of 140 µs every 694 µs within a frame. This sequence is defined by the base PLM firmware installed in the EVM. The 140 µs period corresponds to the time when the hologram is loaded into the device. This does not affect the print because the zero order reflection from the PLM is blocked by the spatial filter in the Fourier plane (see Fig. 1). When a pattern is encoded, the absolute efficiency is close to 24%. With this latter efficiency value, the PLM light engine is approximately 70-fold more efficient than amplitude projections and twice more efficient than DMD holographic encoding previously reported for a holographic engine used in TVAM^13^.

### Holographic projections: computation pipeline

**Fig. 2:**
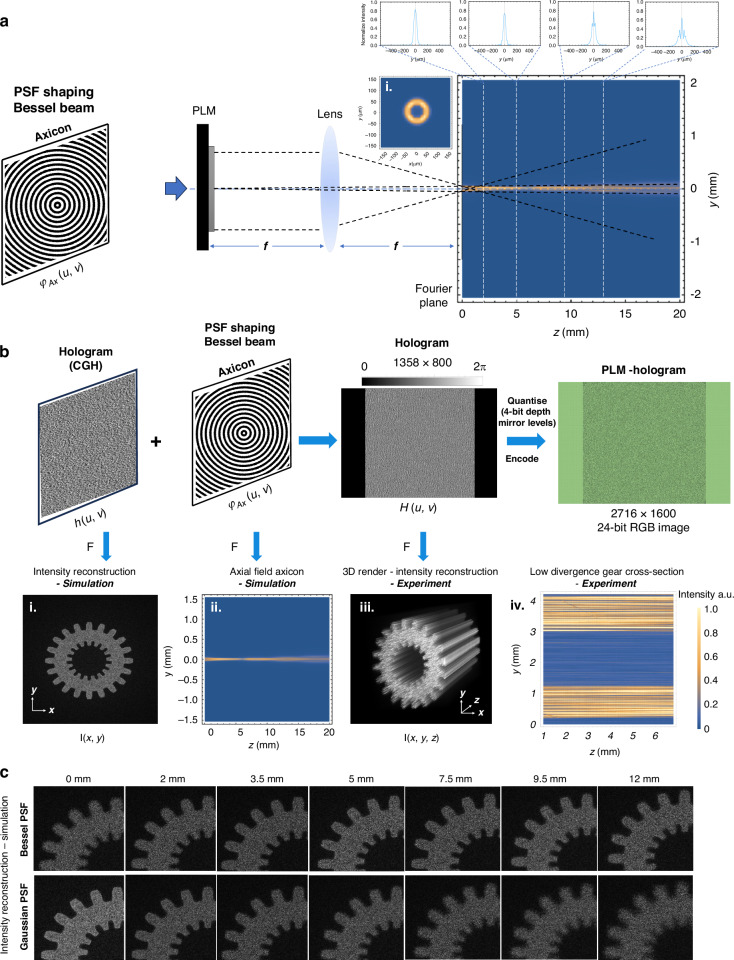
Hologram Computation for printing with PLM. **a** Illustration of the working principle of the Bessel Beam generated with an axicon. i shows the ring intensity distribution in the focal plane (Fourier Plane), which is characteristic of axicons. Profiles of the axial intensity propagation of the axicon are shown on the top where the axicon region starts after $2.5{mm}$ in the near field region. **b** Pipeline for Computing the Holographic Projection $H(u,v)$. A CGH $h(u,v)$ computed by GS Algorithm using an amplitude tomographic projection as the target intensity is convolved with an Axicon phase ${\varphi }_{{Ax}}(u,v)$. i Intensity reconstruction of a gear in the Fourier plane. ii Intensity profile of the axicon along with the propagation direction. iii– iv shows a 3D rendering and cross section of the smeared information when the phase of the gear is convolved with the axicon. **c** Simulation of the intensity reconstruction of a single holographic projection of an extruded gear. Top: Using a Bessel PSF. Bottom: Using an unmodified Gaussian PSF

The phase patterns were computed following the pipeline shown in Fig. 2 (for an extended description, see in Supplementary Note 2). Our technique is based on the tomographic method, where different approaches are used to calculate the amplitude patterns. The Radon transform assumes straight light rays (ray optics). However, this assumption fails at small feature size due to diffraction. A recent theoretical study shows that below approximately $20\,\mu {m}$ feature size, the straight-ray assumption is no longer valid, and wave optics must be considered^24^. In order to achieve a uniform resolution across the print volume in practice, the depth-of-focus of the Gaussian diffraction limited beam is chosen to match the diameter of the vial. In our setup, for example, the resin container has an inner diameter of approximately $11{mm}$, which limits the printable feature to greater than $54\,\mu m$, assuming a Gaussian beam (See further explanation in Supplementary Note 3). To effectively extend this depth-of-focus to fulfill the collimation assumption of the Radon transform, we modify the Point Spread Function (PSF) of the system to form a Bessel beam using holographic projection. The Bessel beam PSF produces a pattern that shows an extended collimated projection length compared to a Gaussian PSF.

In our setup, the printing region is effectively located after the Fourier plane of the PLM. During the hologram pipeline construction process, we first generate an axicon phase ${\varphi }_{{Ax}}(u,v)$, which serves to generate low-divergence beams in the printing region, as shown in Fig. 2a. This axicon phase pattern is then added to the Computer Generated Hologram (CGH) phase pattern $h\left(u,v\right)$ retrieved from the intensity pattern of tomographic projections of the voxelized 3D model^1,10^ using the Gerchberg-Saxton (GS) algorithm. At the Fourier plane of the PLM, the reconstructed wave front is a convolution between the wave front of the CGH $h\left(u,v\right)$ and that of the axion phase ${\varphi }_{{Ax}}(u,v)$. After a short propagation distance beyond the Fourier plane, the reconstructed wavefront forms the desired projection image in which each bright pixel is a Bessel beam that stays focused over a distance much larger than the depth-of-focus of the same image without the axicon, as shown in Fig. 2b (i) - (iv). Once the holographic projection pipeline is created, the stack of phase maps is sent to the PLM control software, which down-samples the phase to 4-bit, encode it properly into video frames for PLM control, and play the frames on the PLM screen. See PLM operation in the Methods section for more detail.

### Speckle evaluation and reduction

Speckle refers to the high-contrast granular interference pattern commonly observed in optical reconstructions of Computer-Generated Holograms (CGHs) displayed on Spatial Light Modulators (SLMs). Essentially, speckle is a result of the coherent superposition of a large number of wave fronts. In our system, the lack of amplitude modulation, the nature of imperfect phase retrieval from the GS algorithm, the pixelation of the PLM, defects in experimental conditions such as dusts and unwanted reflections all contribute to generate a speckle image in the printing region. Speckle in CGHs can be minimized using various techniques, including random phase methods, time multiplexing^24^, tiling holograms^17,25,26^. Recent approaches also explore the use of Neural Networks for time-multiplexed speckle reduction^27^.

**Fig. 3:**
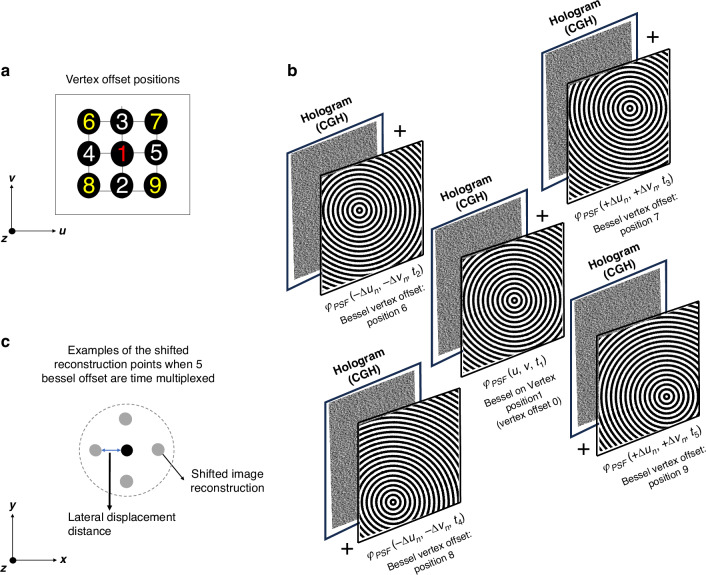
Pipeline to generate a holographic projection with a reduced speckle. **a** Illustration of the 9 different vertex offset displacements used to shift the image reconstruction to a distance shorter than the speckle size. **b** Example of the pipeline used to generate a reduced speckle noise projection. **c** Illustration of the shifted reconstruction points of the holographic projection in the printing plane, when 5 Bessel offset are Time Multiplexed. The displacement is measured relative to the centroid, which is the reconstruction point for the axicon at the original vertex position

Here, we use averaging of spatially shifted reconstructions to effectively reduce the speckle noise. This approach involves time multiplexing up to ${N}_{p}=9$ holograms per projection angle, each representing the holographic projection convolved with one of the 9 axicon phase laterally shifted off-vertex as illustrated in Fig. 3a, b (see details in the method section). The result is a series of projections shifted around the axicon vertex position that, when played sequentially, reduces the speckle noise through the averaging effect in the photopolymerization during printing, which greatly enhances the surface quality of the printed objects. The optimal offset is determined by analyzing the speckle grain size using the power spectrum density (PSD)^28^ of the image of the intensity reconstruction of projected holographic pattern^29^, where the speckle grain size is $43.42\,\mu m$ (See Supplementary Note 4).

Three different offset groups were used. A series of axicon phase patterns define each offset group shifted laterally from the central vertex in 9 different positions, as shown in Fig. 3a. When the resulting holograms are displayed sequentially (time-multiplexed), statistically some peaks of the speckles will coincide with some valleys, reducing the contrast of the speckle intensity compared with a single projection.

The level of speckle reduction is analyzed with the speckle contrast coefficient, a commonly used metric to quantify the speckle level of an image^28–30^.

1 $$c=\frac{{\sigma }_{I}}{\left\langle I\right\rangle }$$

where ${\sigma }_{I}$ is the standard deviation of the image intensity and $\left\langle I\right\rangle$ is the mean image intensity.

**Fig. 4:**
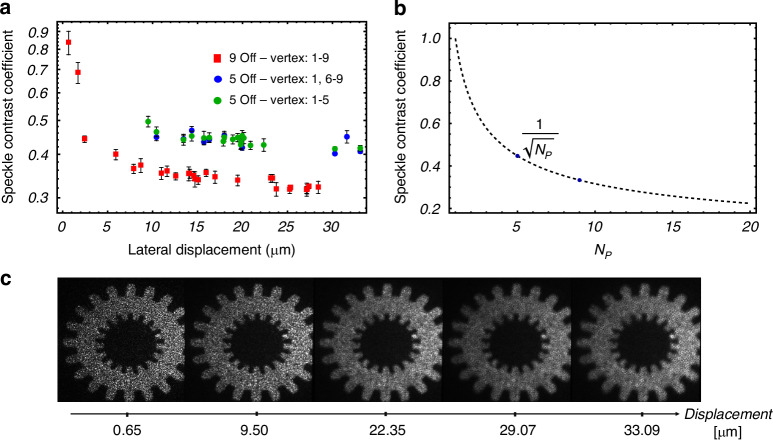
Evaluation of the speckle noise reduction technique. **a** Measured speckle contrast coefficient versus hologram lateral displacement. Error bars represent the standard deviation. Fig. S4 in Supplementary Information shows histogram examples of the images used for the measurement. We did not perform experiments for the vertex offset between 24μm and 30μm for ${N}_{p}=5$ **b** Theoretical speckle contrast coefficient variation related to the number ${{\rm{N}}}_{{\rm{p}}}$ of hologram displayed. **c** Examples of accumulated Intensity reconstructions of a gear when 9 different shifts on the axicon phase produce a lateral shift in the image plane. A plot of the profiles of the gear teeth from the images in ‘c,’ corresponding to different lateral displacements of the holograms, is shown in Fig S5, see Supplementary Information

Figure 4a shows the speckle contrast coefficient as a function of the axicon displacement distance from $1\,\mu m$ to$\,33\,\mu m$ with different number of holographic projections ${N}_{p}$. The speckle contrast coefficient reaches a minimum when the lateral displacements are approximately half the speckle grain size, at which the likelihood of the speckle peaks in one image coincide with the valleys in another is maximized. This trend is statistically confirmed in Fig. 4b. Specifically, for ${N}_{p}=5$, the minimum speckle contrast coefficient is $c=0.45$, while for ${N}_{p}=9$, the minimum possible value is $c=0.33$. These results indicate that the optimal displacement distance, relative to the speckle grain size effectively reduces speckle contrast. From Fig. 4c, we can observe that the intensity reconstruction appears smoother, less grainy when the displacement is close to half the speckle grain size, expecting that, for printing, the surface quality of the printed objects should improve.

### Multi-scale printing with speckle-reduced holographic projections

**Fig. 5:**
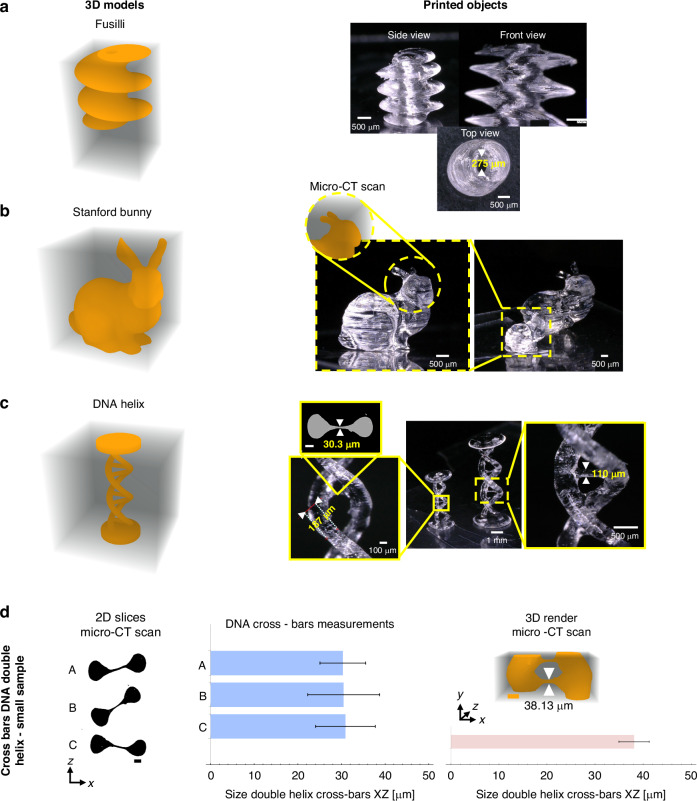
Examples of printed objects with holographic VAM using PLM. **a** Left, 3D CAD model of a Fusilli. Right, printed part on acrylate-based resin of a Fusilli using the speckle noise reduction technique, Printing time: 32 seconds and 18 mW **b** Left, 3D CAD model of the Stanford Bunny. Right, Big and Small models of a Stanford Bunny Printed with acrylate. The big model was printed in 61 seconds, using a laser power output of 50 mW. The small model was printed in 38.46 seconds, using a laser power output: 20 mW. Inset shows a micro-CT scan reconstruction of the bunny where the ears clearly appear**. c**. Left, 3D CAD model of a DNA Helix. Right, Big and Small models of a DNA helix printed with acrylate. Scale bars: $1{mm}$. Inset-left: zoomed-in view of the small DNA helix model. A micro-CT slice shows the smallest positive feature printed. Scale bars: $100\,\mu m$ **d** DNA helix small-model highlights. Left: 2D micro-CT slices through three cross-bars of the same sample (scale bars: $100\,\mu m$); Center: bar chart of the three cross-bar measurements from the printed object (error bars represent standard deviations); Right: 3D micro-CT rendering of one cross-bar, with measurements illustrated in the bar chart (error bars represent standard deviations). Fig S8, see Supplementary Information

To demonstrate the capabilities of our PLM-based light engine for volumetric printing, we successfully fabricate multi-scale objects using holographic projections and the speckle reduction approach described above. Three different photosensitive resins were used in the prints: one acrylate and two hydrogels. First, we used a commercial polyacrylate resin with Diphenyl (2,4,6-trimethylbenzoyl) phosphine oxide (TPO) as the photoinitiator (PI) at a concentration of 1 mM. Thanks to the efficient light engine, we fabricated objects at multiple scales through digital scaling of the holographic projection, as shown in Fig. 5. Figure 5a shows a $4{mm}$ high fusilli 3D model printed in 32 seconds using a laser output power of 18 mW. Figure 5b shows a large and small Stanford Bunny model. The large model is $8{mm}$ high and printed in 61 s with 50 mW laser output power, while the small model is $4{mm}$ high and printed in 38.5 s using 20 mW laser output power. Figure 5c shows a large and small model of a DNA double helix. The small model was printed in 23 s using 20 mW laser output power. The zoomed inset of the large and the small models shows the good surface quality achieved with the speckle reduction technique. Figure 5d shows highlights from the small-size DNA helix model, including the smallest positive feature printed, a DNA helix cross-bars measuring $\sim 30.3\,\mu m$ (XZ) and $\sim 38.13\,\mu m$ laterally (XY). The targeted lateral feature was $\sim 39.9\,\mu m$. In contrast, the lateral (XY) dimension of the larger sample measured$\sim 47.15\,\mu m$, versus the expected $\sim 53.2\,\mu m$. Fig. S8 in Supplementary Information illustrates these measurements with standard deviations, expected values, and model dimensions. The size difference arises from oxygen diffusion, which typically limits sub-$100\,\mu m$ features in photocurable resins. Only three cross-bars remain in the illustrated model, the others broke during post-processing due to their small size, fragility, and oxygen diffusion effects. We also calculated Jaccard indices between micro-CT scans and the model. Values of 0.83 for the large model, and 0.66 for the small model were encountered (see Supplementary Note 8, Fig S8.1). This difference reflects oxygen diffusion’s stronger impact on small-scale samples.

**Fig. 6:**
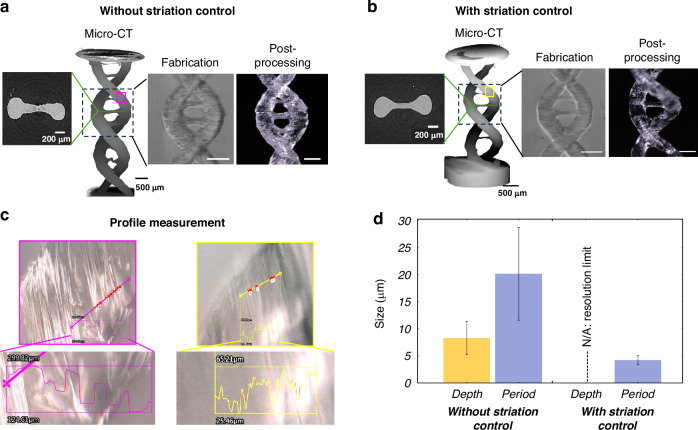
Surface quality improvement comparison. **a** DNA double helix printed without the speckle noise reduction technique. Center: Micro-CT scan of the printed sample. Scale bars: 2$00{\rm{\mu }}{\rm{m}}$. Right: Zoom-in of the printed sample during fabrication, embedded in resin (image from inspection camera 1), and after post-processing. Scale bars: $500{\rm{\mu }}{\rm{m}}$. Left: Representative 2D slice from the micro-CT scan used to measure stria depth. Scale bars: 2$00{\rm{\mu }}{\rm{m}}$. The magenta square indicates the area where the profile measurement was performed for measure the stria width. **b** DNA double helix printed with the speckle noise reduction technique. Center: Micro-CT scan of the printed sample. Scale bars: 2$00{\rm{\mu }}{\rm{m}}$. Right: Zoom-in of the printed sample during fabrication, embedded in resin (image from inspection camera 1), and after post-processing. Scale bars: $500{\rm{\mu }}{\rm{m}}$. Left: Representative 2D slice from the micro-CT scan used to measure stria depth. Scale bars: 2$00{\rm{\mu }}{\rm{m}}$. The yellow square indicates the area where the profile measurement was performed for measure the stria width. **c** Profile images of the area where the stria width was measured, showing examples of the measurement region. **d** Depth and width of the striations measured. Error bars indicate the standard deviation

Figure 6 shows a DNA double helix printed using holograms without and with the speckle noise reduction technique. Figure 6a shows a photo of the printed object without the speckle noise reduction technique. Figure 6b shows a photo of the printed object printed with the speckle noise reduction. The granular pattern of the speckle introduces intensity gaps between the bright grains, which are visible in images captured by inspection camera 1. The application of the speckle noise reduction technique results in visibly smoother surfaces. With reduced granularity in the holograms, there are fewer intensity gaps between the bright grains of the speckles, which in turn diminishes the formation of filaments, due to the self-focusing effect during the polymerization process, that can later cause delamination in the printed objects. We also calculated the Jaccard index for the samples shown in Fig. 6. Values of 0.83 and 0.80 were found for samples with and without stria control, respectively. These results show that stria control not only improves surface quality but also enhances the overall fidelity of the sample (see Supplementary Note 8 and Fig. S8.1).

**Fig. 7:**
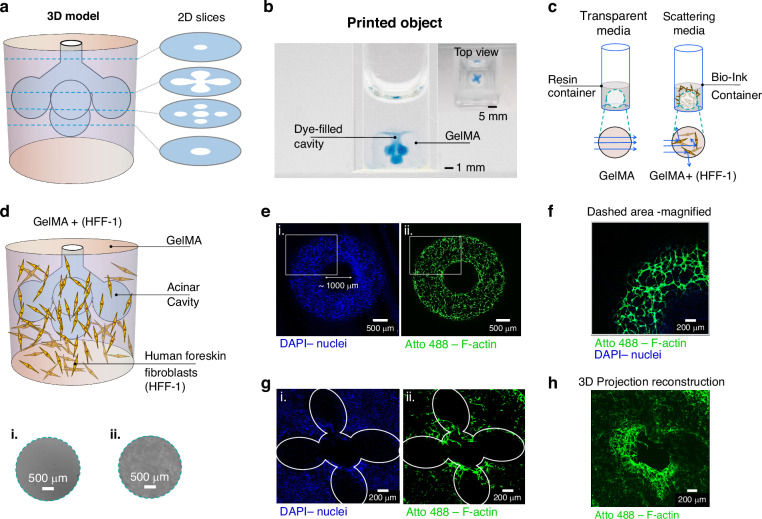
Examples of 3D printed hydrogel constructs. **a** 3D model of the multiacini design with representative 2D slices of the construct displayed. **b** Photograph of the 3D bioprinted construct immersed in water, with the cavity filled with blue dye for visualization. Scale bar: $1{mm}$. The top view highlights the acinar cavity feature. Scale bar: $2{mm}$. **c** Schematic illustration of a resin container showing the appearance of transparent resin compared to a cell-laden hydrogel solution. Light scattering induced by suspended cells within the hydrogel is illustrated. **d** Illustration of the bioprinted construct after printing, showing embedded human fibroblasts distributed around the multiacinar cavity. (i.) Image of the resin container before printing using transparent resin, captured with inspection camera 1. Scale bar: 500*μm*. (ii.) Image of the resin container before printing using cell-laden hydrogel, captured with inspection camera 1. Scale bar: 500*μm*. **e–h** Representative fluorescence confocal images of the bioprinted constructs showing fibroblast networks surrounding the multiacinar cavities. The hydrogels were printed using a laser power output of 55 mW with a printing time of 52 s. **e** Confocal slices corresponding to the inlet region at the top surface of the construct, with nuclei stained in blue (i) and F-actin in green (ii). Scale bars: $500\,\mu m$. **f** Magnified view of the region indicated by the square in (**e**). Scale bar: $200\,\mu m$**. g** confocal slices corresponding to the central region of the construct containing the acinar cavities, with nuclei shown in blue (i) and F-actin in green (ii). Scale bars: 2$00\,\mu m$. **h** Three-dimensional reconstruction of multiple confocal slices highlighting the fibroblast network, with F-actin shown in green along the z-axis. Scale bar: $200\,\mu m$

A gelatin methacryloyl (GelMA) hydrogel laden with human fibroblasts (HFF-1) was used to demonstrate the printing capability of holographic VAM using a phase light modulator (PLM) in scattering resins for biofabrication. A 3D model corresponding to a multiacinar construct (Fig. 7a), designed to mimic the tubuloacinar structures of the exocrine pancreas^31^ was printed^13^. Unlike previous reports using the holographic VAM method^13^, the samples in this study were printed with Bessel beams in a hydrogel containing twice the cell concentration (1 × 10⁶ cells mL⁻¹; see Materials and Methods). In addition, the printed constructs had an approximately 8-fold larger volume, resulting in structures that were about twice as large in each dimension ( ~ 4 mm × 4 mm × 4 mm) compared with those previously achieved. The printed hydrogel constructs closely matched the target geometry (Fig. 7a, b). Filling the internal cavities with blue dye enabled direct visualization of the acinar features and verified the continuity and accessibility of the internal lumen (Fig. 7b). To illustrate the challenging optical environment during printing, we compared the appearance of the resin container filled with transparent resin (non-scattering media) and with a cell-laden hydrogel solution (scattering media), Fig. 7c. Real images from inspection camera 1 shows transparent resin appeared optically homogeneous (Fig 7d–i), the presence of cells within the hydrogel introduced visible light scattering (Fig 7d-ii).

Following printing, fluorescence confocal microscopy was used to visualize cell organization and cytoskeletal structure within the printed constructs (Fig. 7e–h). Fibroblasts exhibited an elongated morphology throughout the full depth of the constructs.

**Fig. 8:**
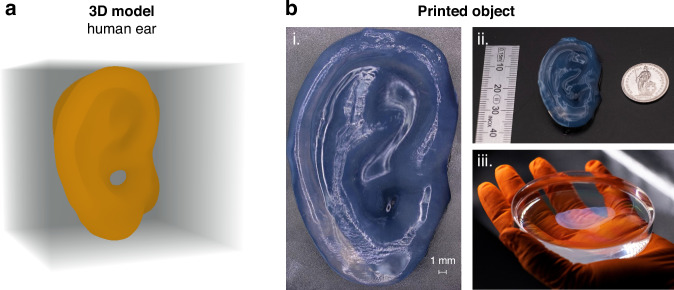
Example of a large-scale 3D object printed in Gelatin Thiol/Norbornene with holographic VAM using PLM. **a** 3D model of a Human Ear. **b** (i.) Photography of a 2D stitching composition of the printed sample after postprocessing. A 3D imaging profilometry result and an example of the holographic patterns used for printing the model are shown in Supplementary Note 7 Fig. S7.2 (ii.), photography of the printed sample after post processing, (iii.) sample of a Human ear model printed with Gelatin thiol/norbornene resin immerse in Phosphate Buffered Saline (PBS) solution. The Human Ear model was printed in 2 min and 12 s, using a power laser output of 150 mW. Supplementary Movie 1 shows the hologram sequence used for printing this model

To illustrate the potential enabled by the improved light efficiency of the light engine, we built an additional setup to print large-scale models. This time, the system magnification is 9 X, allowing us to print structures up to 3 cm×3 cm x 4 cm (the schematic of the experimental setup is shown in Fig S6, Supplementary Note 6). The setup uses the same PLM and light source. Figure 8 illustrates an example of a large-scale sample printed with gelatin Thiol/Norbornene (Gel-SH/NB), and Lithium phenyl-2,4,6-trimethylbenzoylphosphinate (LAP) as photoinitiator (see Materials and Methods). The sample was printed using the speckle noise reduction technique described previously to achieve better surface quality and prevent delamination. The printing time for this large-scale Human Ear sample was 2 min and 12 s, using an output laser power of 150 mW at 405 nm. Gel-SH/NB is known to be much more reactive than GelMA at the same concentration of photoinitiator, offering better structural definition and enhanced resolution thanks to its lower sensitivity to oxygen diffusion compared to conventional (meth)acrylate-based resins. The light engine’s efficiency is clearly demonstrated, since to print objects of size 1 cm^3^, conventional TVAM methods use light sources with power around 6 W^1,10^. Moreover, similar scale samples were printed before using the Helical TVAM^32^ method, for which the used light-source was 1.8 W, and the printing time using acrylate-based resin was up to 10 minutes. For comparison, the same Human Ear model was printed with commercial acrylate resin on the same scale (3 cm × 3 cm × 4 cm), which took 7 min and 45 seconds (see Supplementary Information Fig. S7.2), where thanks to the speckle noise reduction technique the surface quality is smoother showing transparency. We also printed the same model at a different scale (1.5 cm × 1.5 cm × 2.5 cm) by scaling down the target intensity for the calculated holographic projections, completing the print within 5 min (see the printed sample in Supplementary Note 7 Fig. S7.1).

## Discussion

In this work, we have demonstrated the potential of using the new MEMS-based, fast, phase-only modulator (PLM) in Holographic TVAM, which is capable of modulating light at a frame rate up to 1440 fps for holographic printing, marking a significant leap in holographic projection technology. By integrating the PLM, we achieved 70-times more efficient light engine for HoloVAM, enabling faster print times, high-fidelity printed parts and scale from 1 mm^3^ to 36 cm^3^ in a few minutes.

We presented the software control scheme for the DLP6750 PLM EVM. It is implemented in an in-house MATLAB program that quantizes the phase sequence to 4 bits, encodes it into display frames for PLM control, and plays the frames at a constant rate without loss. We also developed a characterization method to measure and calibrate the PLM phase response. Accurate mapping of phase retardance to the corresponding 4-bit voltage bias improves the efficiency of holographic reconstruction. We also introduced a computational pipeline to calculate the holographic projections. We also mitigated the effect of the speckle by using a time-division multiplexing method where a series of grouped CGHs per angle convolved with up to 9 different PSF off vertex allows us to improve the light dose while reducing the speckle noise.

Our work shows that using a PLM in holographic 3D printing enables fast fabrication of multi scale objects using low-power, cost-effective light sources. Furthermore, we demonstrate this approach with diverse materials, including acrylate-based resins, soft hydrogels, and cell-laden hydrogels.

Our speckle-reduction method achieves prints with smooth surface quality, comparable to blurred tomographic printing^33^ implemented with LED light sources. LED-based VAM typically relies on diverging illumination (and associated optical aberrations) to suppress striation artifacts, making them particularly well suited for the fabrication of smooth, continuous geometries such as lenses. However, the achievable construct size is constrained by both pattern efficiency and the limited optical power available from LEDs. The diverging beams also impacts the attainable spatial resolution. In this work, we observed that time-multiplexing axicons to laterally shift the projection patterns leads to a pronounced surface smoothing effect compared to conventional laser-based amplitude TVAM. This approach was initially introduced as an alternative strategy for speckle-noise reduction and was subsequently found to also reduce striation artifacts. This behavior is distinct from previously reported HoloVAM implementations, in which the speckle-noise reduction strategy (HoloTile) resulted in the generation of pronounced striations.

In addition, we experimentally observed that using our holographic light engine together with a Bessel point spread function (PSF), enables printing in scattering media without the need to pre-compensate light patterns, as required in conventional TVAM, where characterization of each individual sample is necessary. We believe that the self-healing properties of the Bessel beam may contribute to this effect. Our method offers a clear advantage, eliminating the need for correction or characterization of each sample as required in conventional TVAM.

Experiments involving cells embedded in hydrogel formulations further confirm the feasibility of this approach for biofabrication applications. Notably, we successfully printed cellularized constructs substantially larger than those previously achieved using earlier HoloVAM strategies¹³, despite the increased light scattering associated with higher cell densities. This demonstrates the advantage of phase-only modulation for holographic printing in optically heterogeneous and scattering media, where conventional holographic approaches may suffer from reduced reconstruction fidelity.

Confocal fluorescence imaging performed six days after printing revealed well-defined fibroblast networks surrounding the multiacinar cavities. These observations confirm that neither the printing process nor the speckle-reduction strategy adversely affected cell viability. Moreover, the close agreement between the confocal images and the representative virtual slices shown in Fig. 7 indicates that the printed geometry is largely preserved over time, even though cellular remodeling may induce minor shape changes.

We investigated the performance and advantages of a hologram-based light engine for tomographic volumetric additive manufacturing (TVAM) using a phase light modulator (PLM). The system enables the fabrication of multiscale objects through digital scaling and enhanced light efficiency. Using a laser diode with a power output of up to 150 mW allowed us to print large-scale samples. $(3\,\times 3\times 4\,{{\rm{cm}}}^{3})$. Recently, Zhan et al.^34^ demonstrated the printing of multiscale, large-volume structures via the incorporation of an amine additive in the resin which reacts with oxygen-derived peroxy radicals to regenerate active propagating radicals. We have demonstrated that incorporating materials following the same photochemistry as Zhan et al., such as Gel-norbornene, enables printing of large samples.

Nevertheless, we believe that improving projection fidelity in the Holographic TVAM technique remains essential. In this work, we enhanced pattern fidelity and light efficiency in the holographic VAM light engine by integrating a 4-bit (16 levels) phase SLM instead of the 2-bit modulator (DMD) used previously. However, the PLM still introduces phase quantization errors and pixel dependent voltage to phase that affect diffraction efficiency and pattern fidelity. These limitations highlight opportunities for future refinements, such as incorporating machine learning-based methods and camera in the loop approaches^35^.

To summarize, we believe that print quality limitations arise from both optical pattern projection fidelity and chemical inhibition. To obtain a more accurate 3D print fidelity, future work requires both to improve the holographic projection and importantly including a model that includes inhibition kinetics. The DNA helix cross-bars and bunny ears (Fig. 5) exemplify that oxygen quenching plays an important role in small structures.

We believe that incorporating oxygen diffusion in the holographic projection patterns optimization will further improve print fidelity for features <50 μm. Other complementary solutions include the use of oxygen scavengers, resin additives, and gel-norbornene-like chemistries. Combined optical–chemical strategies will enhance quality while preserving the advantages of phase encoding.

## Materials and methods

### Experimental setup for 3D printing

The schematic of the optical setup for holographic tomographic additive manufacturing (HoloVAM) using a PLM as a spatial light modulator is shown in Fig. 1a. A fiber-coupled continuous wave (CW) 405 nm blue laser diode (Integrated Optics, 0405L-13A-NI-AT-NF) is used as the light source. This beam is collimated using an aspherical lens (CL) and then obliquely incident upon the PLM arranged in a Fourier configuration with the Fourier lens L1 (focal length f1 = 180 mm), and a spatial filter (SF) is used to filter out the zero-order diffraction at the Fourier plane of L1. Lenses L2 (f2 = 150 mm) and L3 (f3 = 200 mm) form a 4-f system that conjugates the Fourier plane and rescales the holograms by 1.33 X in the printing plane (CP*, Conjugate Fourier Plane). An index matching bath (IMB) is used to reduce the optical distortion caused by the curved interface of the glass vial. For large-scale objects the 4-f system is rescales by 9 X, using L2* (f2* = 40 mm) and L3* (f3* = 360 mm), see Supplementary Note 6.

A rotary stage (RS, Zaber- RSW60C-E03T7-KX13A) holds the sample vial (GV, glass vial), which is set to rotate at a constant speed of 30°/s when the PLM is using HDMI or 60°/s when DP is used. Because of the two different PLM frame rate possible settings, a different time per turn was set when the PLM interface was changed; when using the HDMI interface, the time per rotation is 12 seconds, and when using DP interface, the time per rotation is 6 seconds. Using the in-house MATLAB software, the PLM displays a sequence of holographic projections every ∆θ = 0.5° at a frame rate of 720 Hz (HDMI) or 1440 Hz (DP), corresponding to light dose time per angle of $\sim 16{ms}$ and $\sim 8{ms}$, respectively. The Trigger 2 output of the PLM is used as a counter to ensure precise synchronization between the rotation platform and the hologram sequence. A computer laser control program and a mechanical shutter are used to allow the laser to irradiate the resin sample at a specified power and for a given period. An index-matching bath of vegetable oil (*n* = 1.48) is used to mitigate the lensing effect caused by the cylindrical vials containing the photoresin when the acrylate-based are use, and water (*n* = 1.33) when hydrogels are used.

Two inspection systems were incorporated to monitor the polymerization process and the holographic projections. The first system use a Red LED (R-LED) collimated using a collimation lens (CL) (polymerization monitor) employs lenses L4 (f4 = 100 mm) and L5 (f5 = 180 mm) with camera 1 (C1, iDS UI307xCP-M), while the second system (projections monitor) uses lenses L6 (f6 = 75 mm) and L7 (f7 = 100 mm) with camera 2 (C2, iDS UI327xCP).

### PLM operation

**Fig. 9:**
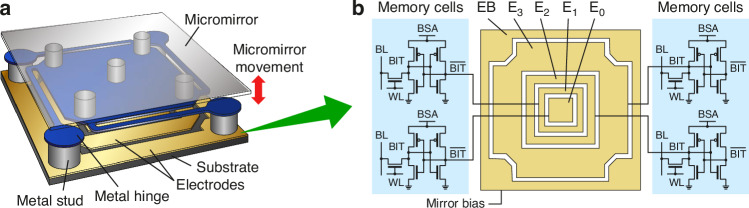
Operational principle of the Texas Instruments’ MEMS-based PLM. **a** Illustration of the mechanical structure of the PLM pixel adapted from the PLM User Guide. **b** Structure of the electrodes and their driving circuitry. Note the inversed bits for E0 and E1. Bit lines (BL), word lines (WL). Note that E0 and E1 bit are inverted

The Texas Instruments PLM is a phase-only SLM based on vertically moving micromirrors. The DLP6750 PLM EVM used in this work features a 1348 × 800 pixel array with a pixel pitch of $10.8\,\mu m$ and uses a video interface (HDMI or DP) for data communication with the host computer. Figure 9 shows the operational principle of the PLM micromirror. The mechanical structure of the PLM pixel is shown in Fig. 9a. The micromirror (grey) sits on the metal hinge (blue), which is maintained at the voltage of the bias voltage electrode (EB) on the substrate. Four control electrodes (E0 – E3) can be switched on or off to a control voltage. The potential difference between the control and the bias electrodes provides an electrostatic attraction force that is programmable depending on the number of control electrodes that are switched to the on state, causing the movement of the micromirror (red arrow). Consequently, the position of the micromirror modulates the phase of the reflected light. Figure 9b shows the structure of the electrodes and their driving circuitry. The micromirror actuation is controlled by four memory cells that are each connected to one of the control electrodes, the content of which is distributed from a computer through either a display interface or a universal serial bus. Owing to the memory cells, the PLM possess an important advantage of no flickering over liquid-crystal based SLMs. Furthermore, the PLM device is polarization insensitive, and adapting it to work with different wavelengths can be simply achieved through adjusting the bias voltage without the need for recalibration.

**Fig. 10:**
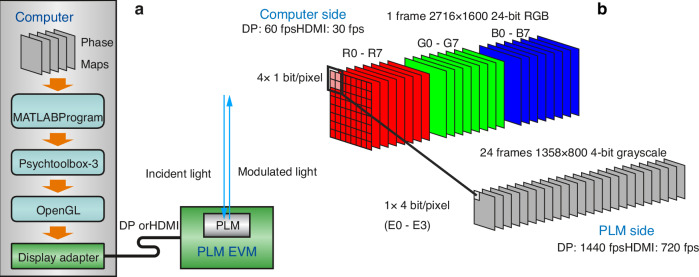
Working scheme of the control software for the DLP6750 PLM EVM. **a** Architectural layering of the control software. An in-house MATLAB program receives the phase sequence to be displayed on the PLM, resamples it to 4-bit, and encode it into computer display frames. Under the assistance of the PsychToolbox-3 package and OpenGL in the operating system, the display frames are sent to the PLM EVM through the display adapter and the DP or HDMI cable. **b**. Bit mapping from the computer display frame to the PLM. From the computer perspective, each display frame contains a bitmap of 2716 × 1600 pixels, each of which is 24-bit red-green-blue (RGB) data encoded in 8 bit per color. From the PLM perspective, on the other hand, each phase map contains 1358 × 800 pixels, each of which is 4-bit grayscale data providing 16-level phase modulation. The control electronics in the PLM EVM treats each incoming display frame from the computer as 24-planes of one-bit bitmap and bins 2 × 2 pixels of each plane into one 4-bit pixel data (E0 – E3) for PLM phase control. The PLM EVM can receive video data at 30 fps through HDMI or 60 fps through DP. Therefore, during the time of one video frame, 24 phase maps are displayed on the PLM, achieving 720 Hz (HDMI) or 1440 Hz (DP) of phase modulation rate

Figure 10 shows the working scheme of the control software for the DLP6750 PLM EVM. The architectural layering of the control software is shown in Fig. 10a. Once connected to a host computer through HDMI or DP, the PLM functions as an additional display screen of 2716 × 1600 pixels on the computer side after basic configurations using a USB-based control software. The bit mapping from the computer display frame to the PLM EVM is illustrated in Fig. 8b. The actuation of each PLM pixel is controlled by four pixel bits from a 2 × 2 pixel block in the corresponding 24-bit RGB display frame received from the computer video interface. This allows the PLM to interpret each received display frame data at 2716 × 1600 pixel resolution as 24 frames of 4-bit micromirror positions at 1358 × 800 pixels per frame, achieving a PLM frame rate of 30 × 24 = 720 Hz and 60 × 24 = 1440 Hz with HDMI (30 fps) and DP (60 fps) interface, respectively. The task of the control program is to reverse-encoding from the phase map required to be displayed on the PLM to the computer side so that each phase map sequence can be correctly displayed on the PLM without frame loss. While the GPU-accelerated encoding step is completed offline, playing the encoded frames on the display adapter at the video rate must be performed in real time. While there is no guarantee for response time in a high-level programming language like MATLAB or Python and a time-sharing operation system such as Windows, we achieved nearly loss-free playing of phase maps owing to the support from Psychtoolbox-3 and the double-buffering capability of OpenGL without resorting to C/C + + program and DirectX.

### PLM calibration

The PLM is capable of producing a phase shift of up to 2*π* for a wavelength range of $405{nm} < \,\lambda \, < \,650{nm}$, which is wavelength-dependent. The PLM provides a mirror bias voltage control that is used to adjust the mirror displacement according to the wavelength. The calibration is achieved by measuring this phase delay as a function of the micro-mirror state.

**Fig. 11:**
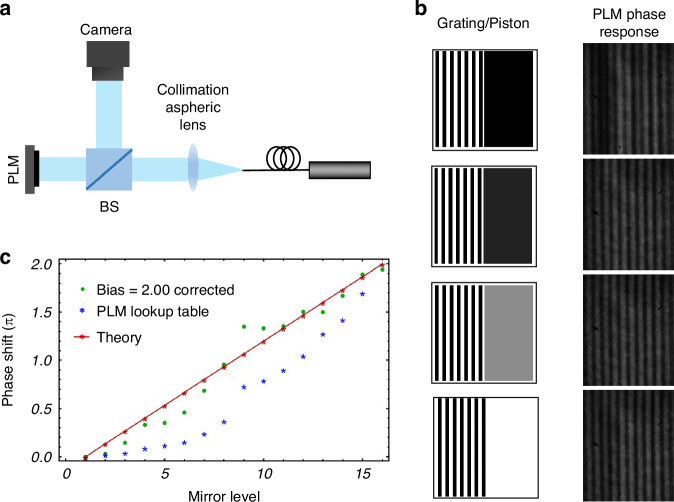
Phase calibration. **a** Phase calibration diagram **b** (Left row) Phase is displayed on the PLM for calibration. (Right row) corresponding fringe patterns**. c** Phase modulation graph relative to the mirror level. Blue represents the lookup table provided by the manufacturer, the red represents the desired linear performance of the phase delay

A calibration was performed for the correct operation of PLM. Several phase calibration methods for phase-only modulators based on LCOS SLM have been reported in the literature. Common methods include phase shift interferometry based on the analysis of interference fringes obtained in a Michelson interferometer^16^, two-beam interferometry^16,21^, binary diffraction grating, and many others based on the Muller matrix and Jones vectors^36–38^. In contrast to LCOS, where the phase delay is based on birefringence and each pixel is a Pockels cell, the operation of the PLM is based on the phase delay imparted for vertical displacements of the micromirror. Therefore, the phase delay in each pixel is a function of the 4-bit response of the mirror position. Our calibration is an adaptation of an interferometric method that uses a self-generated diffraction grating^23^. Figure 11a shows the optical setup used for phase calibration. For this method, the image displayed on the PLM consists of two sectors, as shown in Fig. 11b. One part of the image consists of a binary grating, which is kept constant, while the other part of the image is a uniform gray level that varies. These gray levels correspond to different mirror positions. When a collimated beam is incident on the PLM, the binary grating region diffracts the light, splitting the beam into several diffraction orders, while the uniform gray level region (piston) modifies the optical path of the reflected light. Consequently, the shifts in the resulting interference fringes between the diffracted and the reflected beams is proportional to the induced phase change in the reflected beam, which is used in our calibration.

### Time multiplexing of computer-generated holograms with lateral shift

The lateral shift imparted to the holographic projections is achieved by convolving the CGH phase with axicon phases with different offsets. When these holographic projections are displayed sequentially over time, the accumulated intensity generates a holographic projection with reduced speckle noise, resulting in a smoother image as explained above. However, a small lateral shift produced by an axicon with its vertex off-axis can generate a slight angular divergence in the light intensity distribution during near-field propagation, which is also transferred to the intensity reconstruction of the desired shape when the holographic projection is convolved with an axicon off-axis (See Supplementary Information Note 2. Fig S2.1.a, b, angular shift of the axicon axil intensity).

**Fig. 12:**
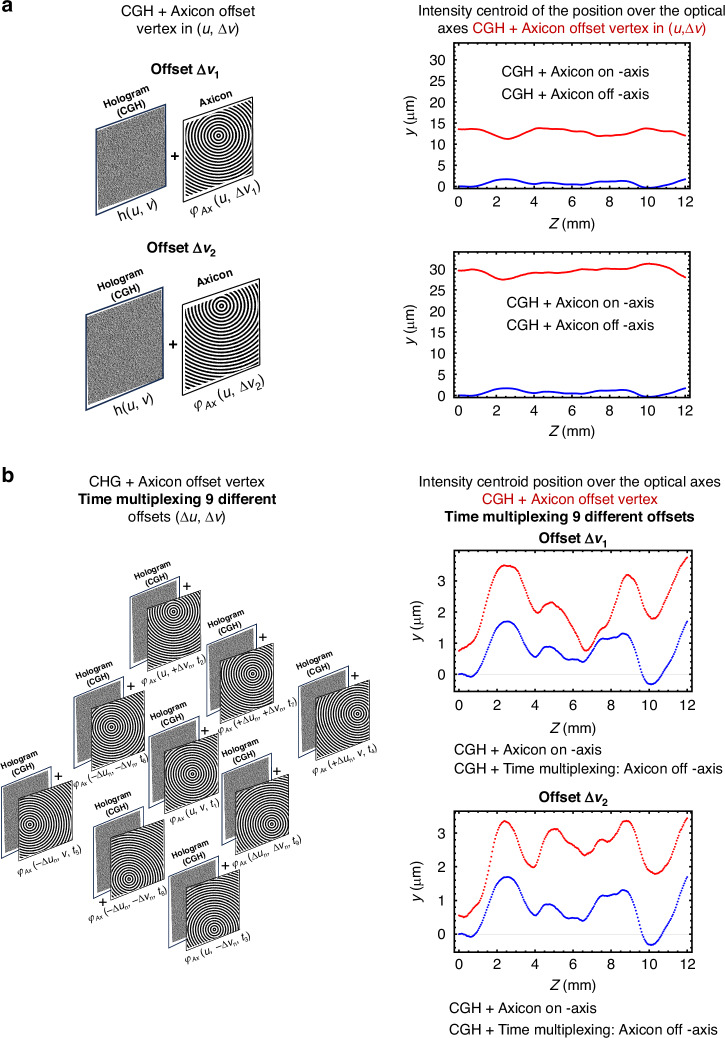
Phase hologram used to generate holographic projections with a lateral shift. **a** Left: Phase hologram of the off-axis axicon convolved with the calculated computer-generated hologram (CGH) of a gear. Right: Plot of the gear intensity centroid position over the axial propagation. **b** Left: Calculated computer-generated hologram (CGH) of a gear convolved with the 9 Phase hologram of the off-axis axicons. Right: Plot of the averaged gear intensity centroid position when the CGH is multiplexed with Axicon offset in 9 different lateral positions

To evaluate the shift imparted to the holographic projection, we determined the intensity centroid position of the reconstructed gear pattern along the propagation distance. The Intensity Centroid is a spatial intensity measurement that provides the coordinates of the intensity-weighted centroid, which we then track over the propagation distance. Figure 12a illustrates two examples of the shifts imparted to reduce speckle noise. The vertex shift generates a lateral displacement of the holographic projection in the y-direction of approximately $\sim 12\,\mu m$ (Fig.12a -top) and $\sim 31\,\mu m$ (Fig.12a -bottom). In both cases, a slight angular divergence of $\pm \,3\,\mu m$ is noticeable along the propagation. However, when the time multiplexing of the holographic projections is performed, the centroid exhibits an average position that propagates parallel to the axicon on-axis, with a divergence of less than 3*μm*. The small fluctuations in the intensity centroid are due to changes in the speckle pattern during propagation, which are influenced by the Bessel intensity distribution of the point spread function (PSF). This effect enables us to achieve prints with smooth surface quality, similar to previous works such as blurred tomographic printing^33^.

### Photocurable resin

#### Acrylate

Photoresin was formulated by combining the photoinitiator TPO (Diphenyl (2,4,6-trimethylbenzoyl)-phosphine oxide, Sigma) with commercial polyacrylate photoresin (PRO 21905, Sartomer) at a concentration of 1 mM. The resin has a viscosity of 20*P s*, at temperature. The mixture of the resin with TPO was homogenized using a planetary mixer deaerator (Kurabo Mazerustar KK-250SE). The photocurable resin was subsequently transferred into cylindrical glass vials (12 mm outer diameter) and subjected to sonication to eliminate air bubbles. For large-scale samples, mixture preparation and formulation were kept similar; however, the resin container had an inner diameter of 30 mm. After transferring the resin to the glass vial, we introduced it to the vacuum chamber until the air bubbles were reduced. Later, the resin container was kept open in the fume hood overnight to avoid inhomogeneities in the sample polymerization.

#### Hydrogels

*Gelatin metacryloyl (GelMA):* GelMA was synthesized from type A porcine gelatin (Sigma, G2500) following the protocol of Van De Bulcke et al^39^. Briefly, the gelatin (10% w/v in Phosphate Buffered Saline (PBS), 50°C) was functionalized with methacrylic anhydride at 50 °C for 3 h, then lyophilized and stored at −20 °C^31^. For hydrogel preparation, lyophilized GelMA was dissolved at 7% w/v in PBS, and lithium phenyl-2,4,6-trimethylbenzoylphosphinate (LAP, Sigma-Aldrich, 900889) was added at 0.5 mg/mL as the photoinitiator. Before use, the photoresin was filtered through 0.2 µm filters both to ensure sterility for subsequent cell-based applications and to remove scattering particles that could interfere with the photopolymerization process.

*Cell culture of HFF-1:* Human foreskin fibroblasts (HFF-1) obtained from ATCC® were cultured in DMEM without phenol red, supplemented with 1% penicillinstre penicillin streptomycin ptomycin, 2 mM L-glutamine, and 15% fetal bovine serum (all Gibco), and maintained at 37 °C in 5% CO₂.

*Gelatin thiol/norbornene resin (Gel-SH / NB):* The synthesis of gelatin-norbornene (Gel-NB) was performed as previously reported, and the degree of substitution was found to be ≈ 0.17 mmol/g^40^. The synthesis of gelatin-thiol (Gel-SH) was performed as previously reported^40^, and the degree of substitution was found to be ≈ 0.28 mmol/g. Freeze-dried Gel-SH and Gel-NB were dissolved at 5% w/v (total polymer concentration) in PBS at 37 °C with 1:1 molar ratio of SH:NB. Photoinitiator lithium phenyl-2,4,6-trimethylbenzoylphosphinate (LAP) was added from a stock solution of 2.5% w/v in PBS to obtain a final concentration of 0.05% w/v. Before use, the photoresin was filtered (0.2 µm filters) to remove debris and scattering particles. The warm resin was poured into cylindrical glass vials (30 mm outer diameter) and left to thermally gel at 4 °C for 15 min.

### Post-processing

#### Acrylate

Once printing was complete, the printed parts were extracted from the glass cylinders and immersed in propylene glycol monomethyl ether acetate (PMGEA) for a 10-minute rinse under gentle stirring using a vortex mixer. Subsequently, they underwent an additional 10-minute cleaning process in isopropyl alcohol (IPA). The printed samples were post-cured under UV for 10 minutes.

#### Cell-laden GelMA

After printing, the glass vial was placed in a water bath at 37 °C for 5 min. In a biosafety cabinet at sterile conditions, a pre-warmed PBS was added to the vial to wash out the unpolymerized GelMA. The cell-laden constructs were transferred to multi-well plates filled with cell medium and kept in the humidified CO2 incubator at 37 °C for 6 days before the imaging session.

#### Gel-SH / NB

Once printing was complete, the vial was placed in an incubator at 37 °C for a few minutes to dissolve the uncrosslinked resin. The printed part was then gently washed in warm PBS, and then postcured for 30 s under UV.

### Confocal imaging of bioprinted constructs

#### Sample Preparation and Staining

Printed hydrogel constructs were permeabilized with 0.1% Triton X-100 in PBS for 10 min at room temperature, followed by three washes of 5 min each with PBS.

To visualize cellular cytoskeletons, samples were incubated with Atto 488 (1:100 in PBS) for 30 min at room temperature, followed by three washes of 5 min each with PBS. Nuclei were stained with DAPI (1:1000 in PBS) for 5 minutes at room temperature, followed by a single PBS wash. All staining steps were performed in wet chambers protected from intense light.

#### Confocal imaging

Samples were imaged using a motorized inverted confocal microscope (Leica SP8). Sequential two-channel fluorescence acquisitions were performed to minimize spectral cross-talk: DAPI fluorescence was collected at 440–480 nm, and Atto 488 fluorescence was collected at 498–542 nm.

Three-dimensional image stacks (z-stacks) were acquired across the full thickness of the printed constructs with an axial step size of 10 μm. Image acquisition and microscope control were performed using LAS X software (Leica). Acquired images were processed and analyzed using Fiji/ImageJ.

#### Micro-CT scans

Millimeter-scale printed objects were imaged with voxel sizes of 3 × 3 × 3 μm3 under a 160 kV X-ray transmission tomography (Hamamatsu, Japan). 3D visualizations and cross sections of the pieces were obtained using Fiji-ImageJ. centimeter-scale sample objects were imaged with voxel sizes of 20 × 20 × 20 μm3.

#### Photography

The printed parts were analyzed using a Keyence digital microscope (VHX-5000) with magnifications ranging from x20 to x200. The Human ear printed with norbornene was imaged using a FUJIFILM camera with an objective with a magnification ranging from x55 to x200, adapted with a MCEX-16 extension tube.

#### Simulations

3D renderings of STL files, along with recorded light intensities and simulated intensity distributions, were generated using Wolfram Mathematica 14.2^41^. The tomographic projections were calculated through the Radon transform (Intensity targets for the holographic projections), as well as CGHs generation were calculated in MATLAB R2025a^42^.

#### 3D models

The 3D Model of The Fusilli is custom-made. The DNA helix by linew on Thingiverse (https://www.thingiverse.com/thing:3980700) and The Stanford Bunny by printable (https://www.printables.com/model/364039-stanford-bunny-fdm-printable/files) are is licensed under CC BY 4.0. The 3D model of the Human Ear by Faaxundo is licensed under CC BY-SA.

## Supplementary information

Supplementary Information for High-Efficiency Multi-Scale Holographic Volumetric 3D Printing with a Phase Light Modulator

Supplementary Movie 1. Holographic Projection Sequence

## Data Availability

The data supporting the results of this study are available in the Supplementary Information.

## Code availability

The code presented in this paper are available upon reasonable request.
