# Supplementary material for: High-efficiency multi-scale holographic volumetric 3D printing with a phase light modulator
Source: Light Sci Appl. 2026 May 19;15:241. doi: 10.1038/s41377-026-02331-4 (PMC13187184; doi:10.1038/s41377-026-02331-4)
Supplement: Supplementary file 1 — Supplementary Information for High-Efficiency Multi-Scale Holographic Volumetric 3D Printing with a Phase Light Modulator [file 41377_2026_2331_MOESM1_ESM.pdf]

Supplementary Information for

High-Efficiency Multi-Scale Holographic Volumetric 3D Printing with a Phase Light Modulator

Maria Isabel Álvarez-Castaño[1], Riccardo Rizzo[1], Viola Sgarminato[1,2], Ye Pu[1],*

*Christophe Moser[1]**

[1]Laboratory of Applied Photonics Devices, School of Engineering, Ecole Polytechnique Fédérale de Lausanne, CH-1015, Lausanne, Switzerland

[2]Present address: Politecnico di Torino

Supplementary Note 1: Light efficiency measurements:

Figure S1 shows the experimental setup used for the volumetric 3D printer based on holographic projections. The setup integrates two light engines: one using a DMD with binary holograms, and another using a PLM with 4-bit holograms. In the previous work by Álvarez-Castaño et. al.[1], the efficiency was measured along the light path indicated in light blue. The amplitude and phase efficiencies for the DMD were reported ass $\eta_{\mathrm{phase}}^{\mathrm{DMD}} = 0.34\ \%$ and $\eta_{\mathrm{phase}}^{\mathrm{DMD}} = 9.71\ \%$, respectively. The measured power corresponds to the amplitude projection and the holographic reconstruction of the same target shape, with the same expected reconstruction size. Similarly, we measured the input power (light incident on the PLM), which was $\mathrm{P_{in}} = 38.03\ \mathrm{mW}$. A computer-generated hologram (CGH) producing the same intensity reconstruction was then displayed on the PLM. Subsequently, the power was measured in the conjugate plane of the Fourier plane (CP*). The output power after the 4F system (printing plane) was $\mathrm{P_{out}} = 9.04\ \mathrm{mW}$, resulting in a pattern efficiency of $\eta_{\mathrm{phase}}^{\mathrm{PLM}} = 23.78\ \%$.

A diffraction grating with a linear carrier of $\Lambda = 4$ were displayed on the PLM, which is the linear phase added to the holographic projections to filter out the zero order of diffraction from the patterns in the printing plane. These measurements give us $45.91\ \%$

$$\frac{I_{=+1}}{I_0} = \frac{2.53\ mW}{5.51\ mW} \tag{S-0}$$

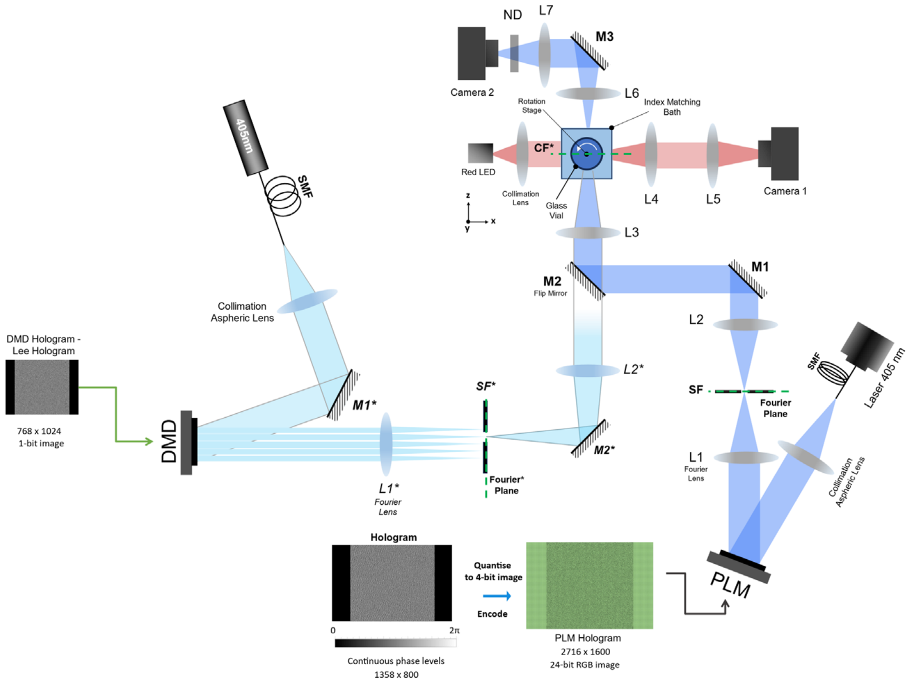

Fig S1. Experimental setup of the Holographic Tomographic Volumetric Additive manufacturing (HoloVAM) using two different light engines. The light blue color indicates the light path trajectory of the light engine that uses a DMD (Vialux DLP7000, 1024 × 768 resolution, pixel size 13.76 μm) as a spatial light modulator. In this configuration, mirror M2 is flipped to direct the light toward the printing plane (CP*, Conjugate plane), which is the conjugate plane of the Fourier plane of lens L1*, formed using lenses L2* and L3 (Green dashed lines). A Spatial Filter (SF*) is placed in the Fourier plane of the L1* to allow the propagation of the order -1 which is the most efficient when using the Lee Hologram method. The darker blue color represents the light path of the light engine using a PLM (TI DLP6750 PLM EVM, 1358 × 800 resolution, pixel size 10.8 μm). For this path, the printing plane is also the Conjugate Plane (CP) of the Fourier lens L1 (green dashed lines), using the lenses L2 and L3. A Spatial Filter (SF).

For comparation, Fig. S1.1 shows the experimental Pattern Reconstruction with the DMD system and PLM.

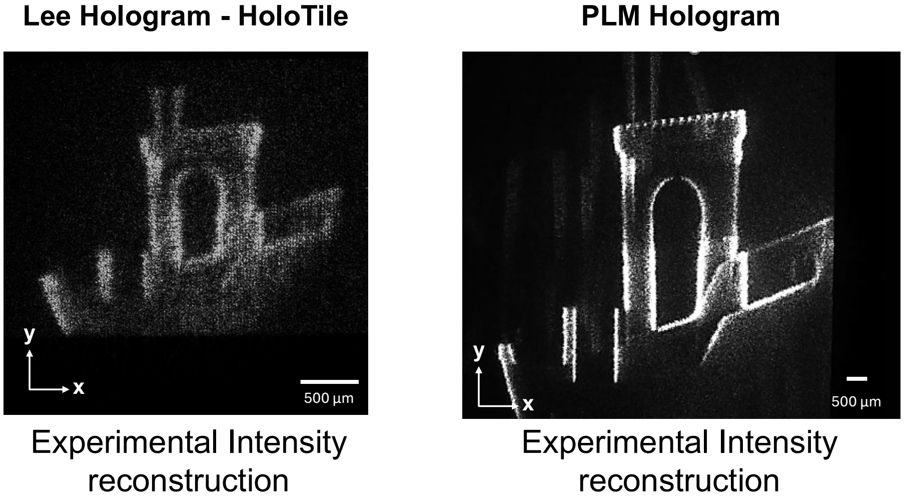

Fig. S1.1. Experimental Pattern Reconstruction Comparison: (left) prior work (binary modulator) vs (right) current work (16 level phase).

Supplementary Note 2: Holographic projection generation – Phase pipeline

The complete pipeline for holographic projection calculation is illustrated in Fig. S2, where the target intensity for our holograms corresponds to the amplitude tomographic projections, which are calculated using the Radon transform and Filtered Back Projections (FBP) [2]. Afterwards, a phase retrieval algorithm, in this case the traditional Gerchberg-Saxton method is used to calculate the final hologram $h(u,v)$ [3], [1].

To fulfill the collimation assumptions of the Radon transform, it is necessary to use a low-divergence beam that extrudes the projection information throughout the entire printing volume. To achieve this, we modify the point spread function (PSF) of the system using a Bessel beam.

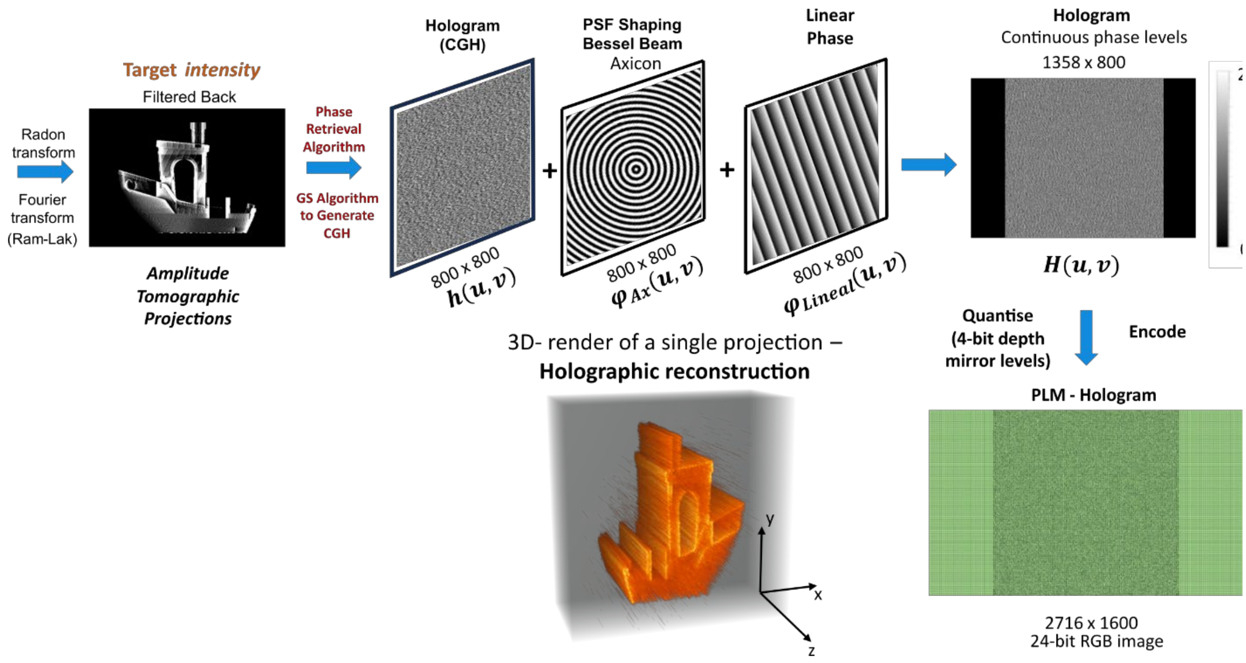

Fig. S2 Complete computation pipeline for holographic projections

Bessel beams are well known because, unlike Gaussian beams, they are non-diffracting beams whose transverse profile is described by a Bessel function, with a central maximum and concentric rings, and remains unchanged during propagation. There are different approaches to generating Bessel beams[4], [5] . For far-field approaches, a ring aperture is used. And for near-field approaches, a conical lens or axicon is used. In this work we are using axicon phases where the mathematical expression to generate the hologram is

$$\varphi_{Ax}(u, v) = Mod[-k\alpha(n-1)\sqrt{u^2+v^2},\ 2\pi] \tag{S-1}$$

Where k is the wave number of the incident beam, n is the refractive index of the axicon, α is the bottom angle of the axicon, and (u, v) is the coordinate with the center of the SLM.

Finally, the blazed grating that separates the first diffraction order from the zero order is

$$\varphi_{Lineal}(u, v) = Mod[2\pi\ G_u u + G_v v, 2\pi] \tag{S-2}$$

Where G_u and G_v are the grating frequencies in the respective u and v directions. Therefore, the final hologram displayed on the PLM, which corresponds to the holographic projection, is expressed generally as

$$H(u,v,\theta) = Mod[\, h(u,v) \; + \; \varphi_{Ax}(u,v) + \; \varphi_{grating}(u,v), 2\pi] \qquad \text{(S-3)}$$

where $\mathrm{h(u,v)}$ is the phase retrieved from the GS algorithm.

Supplementary Note 2.1: Axicon shift

For an axicon with and offset, the phase distribution takes the form

$$\varphi_{PSFshif}(u,v) = Mod[-k\alpha(n-1)\sqrt{(u-\Delta u)^2 + (v-\Delta v)^2},\, 2\pi] \qquad \text{(S-4)}$$

Where Δu and Δv are offset of the hologram's center at (u,v), axicon on-axis. Then the vertex coordinate after the offset is $(\Delta u, \Delta v)$, axicon off-axes. Fig. S2.1a. top row, Illustrates the axicon with its vertex center at (u,v) with its corresponding phase and axial propagation. Bottom row illustrates the axicon with its vertex center at $(\Delta u, \Delta v)$ (off-axis) with its corresponding phase and axial propagation. Fig. S2.1a-b shows the axial light intensity distribution over the propagation, where the axial intensity distribution of the axicon on-axis propagates parallel along the optical axis, while the axial intensity distribution of the axicon off-axis has a small tilt.

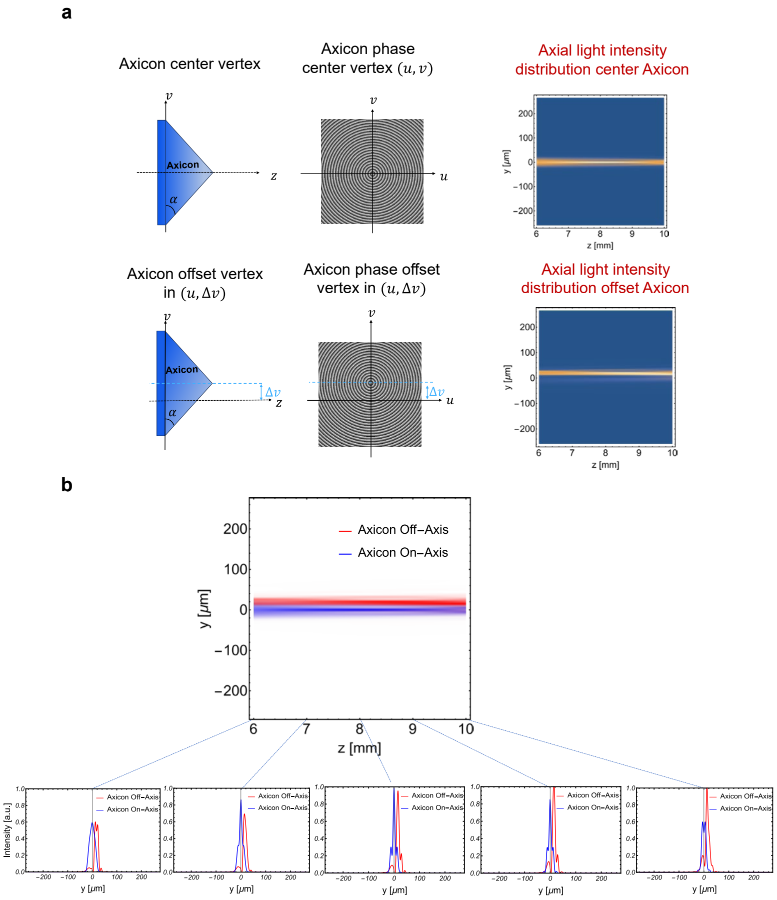

Fig S2.1. a Top left: Phase hologram for generating a Bessel beam with the vertex at the center (axicon on-axis, (u, v). Top right: Axial light intensity distribution of a single Bessel beam on-axis. Bottom left: Phase hologram for generating a Bessel beam with the axicon vertex centered at $(u, \Delta v)$. Bottom right: Axial light intensity distribution of a single Bessel beam generated by an off-axis axicon. **b** Combined axial intensities of the axicon on-axis (blue) and off-axis (red) are shown. Intensity profiles along the propagation direction are displayed

Supplementary Note 3: System resolution

Beam divergence is an important parameter for the tomographic method, as most algorithms used to calculate tomographic projections assume a collimated beam or straight light rays in ray tracing. In practice, light diverges, so divergence limits resolution. Only within the Rayleigh length of the beam can we achieve a homogeneous pixel size throughout the printing volume.

Conventional TVAM uses the divergence calculation analysis, where the Rayleigh range (z_R) relates to the propagation distance along the printing volume (here, vial diameter D). In this case the vial diameter ($2z_R$). The minimal feature size is the beam waist diameter $d = 2\omega_o$. The Rayleigh range is

$$z_R = \frac{\pi\omega_o^2}{\lambda} \qquad \text{(S-5)}$$

where λ is the wavelength, ω_o is the beam waist. For a glass vial with inner diameter of 11 mm, the minimal feature size is

$$d = 2\sqrt{\frac{\lambda * z_R}{\pi}} = 2\sqrt{\frac{(405\ \times 10^{-9}) * (5.5 \times 10^{-3})}{\pi}} = 53\ \mu m$$

To maintain a collimated beam and achieve higher resolution throughout the printing volume, the holographic method offers high flexibility because it is not constrained by a Gaussian PSF. To extend the depth of field, we use a Bessel PSF. A Bessel-like, non-diffracting beam profile is produced using an axicon phase (see Supplementary Note 2). The advantage of modifying the PSF with the axicon phase is that it allows us to design the axial range or Bessel beam length digitally as a function of the beam radius, axicon angle, and axicon refractive index. For a collimated beam of radius R, the length of the non-diffracting region is given by:

$$Z_{max} = \frac{R}{\alpha_{ax}(n_r - 1)} \qquad \text{(S-6)}$$

whereα_{ax} is the axicon angle, and $n_r$axicon refractive index.

Our system is diffraction-limited; the minimal feature size is determined by the system's numerical aperture (NA) in the Fourier plane. We use a PLM in a Fourier configuration and therefore

consider the active area of the PLM as an aperture with dimensions of 800 pixels and a 10.8 μm pixel pitch. Thus, the lateral resolution is

$$d_{xy} \approx \frac{\lambda}{2\,\mathrm{NA}} \tag{S-7}$$

We calculate the NA as

$$NA = Sin[ArcTan\left[\frac{\frac{Aperture}{2}}{f}\right]] \tag{S-8}$$

Where f is the focal distance of the Fourier plane. We can later scale the minimal feature size by considering the optical magnification of the system.

Supplementary Note 4: Power Spectral Density

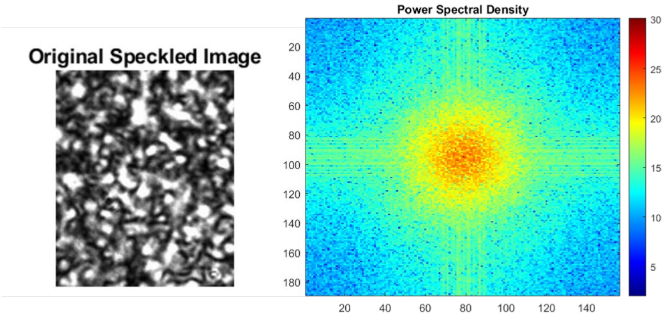

Fig. S3. Left, original speckle image. Right, Power Spectral Density

To implement the method, it is necessary to calculate the appropriate lateral displacement meaning the off-vertex shift required to reduce the speckle noise across the multiplexed holograms. The optimal offset is determined by analyzing the speckle grain size using the power spectrum density (PSD) of the image of the intensity reconstruction of projected holographic pattern. The power spectrum density $P(u, v)$ is

$$P(u,v) = \frac{|FFT\{I(x,y)\}|^2}{L * W} \tag{S-9}$$

Where, FFT stands for Fast Fourier Transform, and L and W represent the vertical and horizontal components of the image. Here, the inverse of the dominant spatial frequency corresponds to the average speckle grain size, which in our measurement is $43.42\ \mu m$. The speckle grain size in our technique corresponds to the vertex displacement. By accumulating the intensity reconstruction over time from N_p holographic projections that are laterally displaced with 'uncorrelated' speckle noise, due to shifts imparted by the PSF (axicon out of the vertex), we expect the speckle contrast to decrease.

Supplementary Note 5: Speckle contrast coefficient measurement

We collected images from the experimental setup within the near-field region to obtain the intensity reconstruction of a gear. During the experiment, we time-multiplexed the CGH phase convolved with nine different axicon phases, which produced different lateral displacements. All images were acquired using the same integration time, with the PLM operating at a frame rate of 1440 fps. To measure the speckle contrast coefficient in the experimental intensity reconstructions, we analyzed different patches in the images, as shown in Fig. S4 (top row), and calculated the speckle contrast coefficient using equation (1).

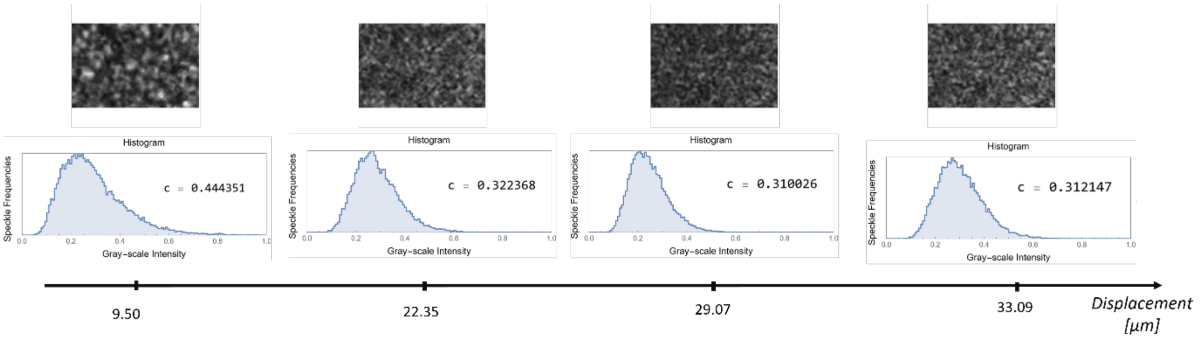

Fig. S4. Speckle Image Analysis. Top row: An example of one of the patches analyzed from a single image. Each column represents a different displacement**.** Bottom row: Histogram distribution of the images with their corresponding speckle contrast coefficients.

As lateral displacement increases, the gap between the maximum and minimum values of the bright grains decreases (see gear teeth profile in Fig S5), which improves the light dose during the printing process, this result will improve the surface quality, and which prevents the printed objects from delaminating.

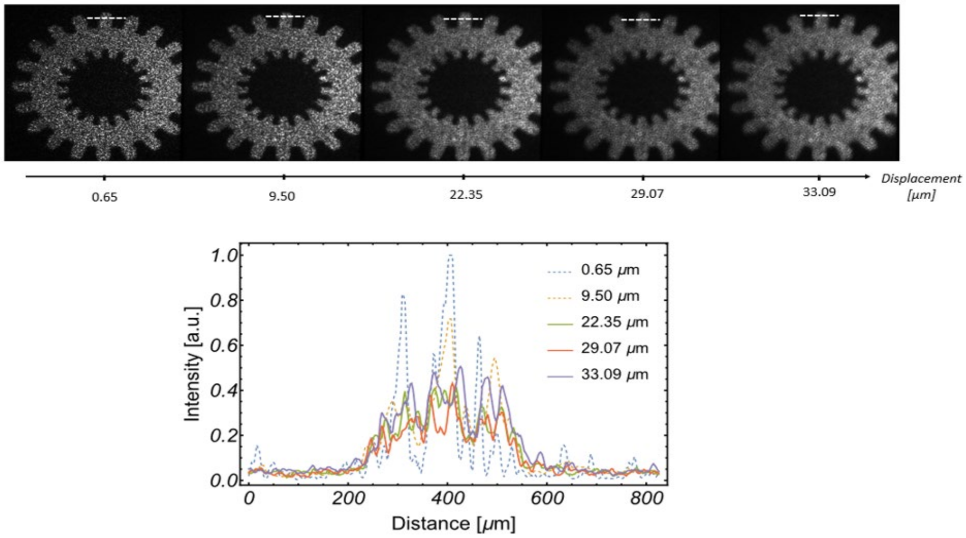

Fig S5. Top: Examples of accumulated Intensity reconstructions of a gear when 9 different shifts on the axicon phase produce a lateral shift in the image plane. Bottom: Intensity profiles of a gear tooth for different lateral displacements.

Supplementary Note 6: Experimental Setup for large scale objects

Figure S6 illustrates the integration of both configurations, enabling the printing of small- and large-scale objects using the same light source and PLM. The setup consists of two light paths corresponding to two different magnifications: 1.33× for small-scale objects and 9× for large-scale objects. Lenses (Flip L2 and Flip L2*) and a mirror (Flip Mirror M1) are mounted in flip mounts, allowing us to switch between configurations.

The blue rays represent the light path for the small-scale configuration (see Materials and Methods), in which the Flip L2 lens and Flip Mirror M1 are flipped in, and the Flip L2* lens is flipped out. The solid blue path represents the large-scale configuration, in which the Flip L2 lens and Flip Mirror M1 are flipped out, and the Flip L2* lens is flipped in. In this case, a 4-f system is formed to conjugate the Fourier plane of lens L1, using lens L2* ($f2^* = 40$ mm) and lens L3* ($f3^* = 360$ mm). Mirror M1* redirects the light path to the printing plane, where a glass vial (GV) of 30 mm diameter contains the photocurable resin.

To monitor the holographic projections, an inspection system was implemented, employing lenses L4* ($f4^* = 50$ mm) and L5* ($f5^* = 25$ mm) to demagnify the holographic projection by 6× and obtain the intensity reconstruction on camera C3.

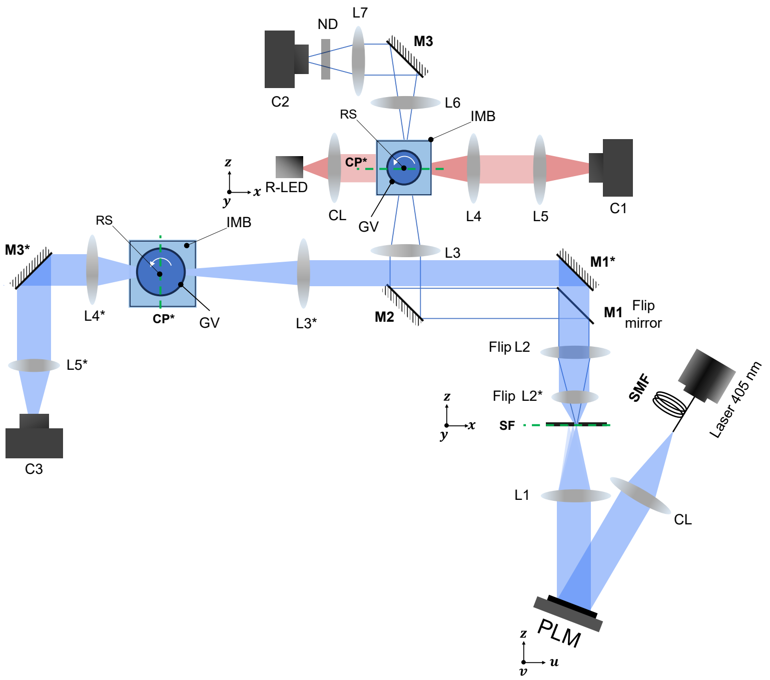

Fig S6. Optical Configuration for Large-scale samples. CL: Collimation Lens, PLM: Spatial Light Modulator (TI DLP6750 PLM EVM, 1358 × 800 resolution, pixel size 10.8 µm), SF: Spatial Filter. L: Lens, M: Mirror, CP: Conjugate Plane, GV: Glass Vials, IMB: Index Matching Bath, RS: Rotation Stage. ND: Neutral Density Filter, C: Camera.

Supplementary Note 7: Large-scale example using acrylate -based resin

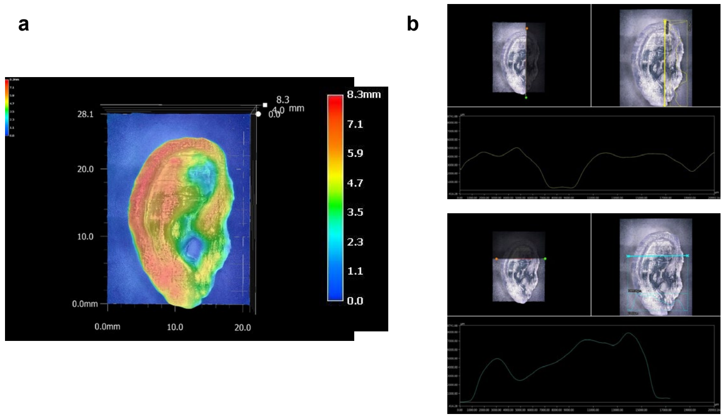

Fig S7. 3D-printed example of a large-scale object in acrylate-based resin with holographic VAM using PLM. a 3D profile Human ear model printed with acrylate. **b** Vertical and horizontal profile of the Human ear model printed with acrylate**.**

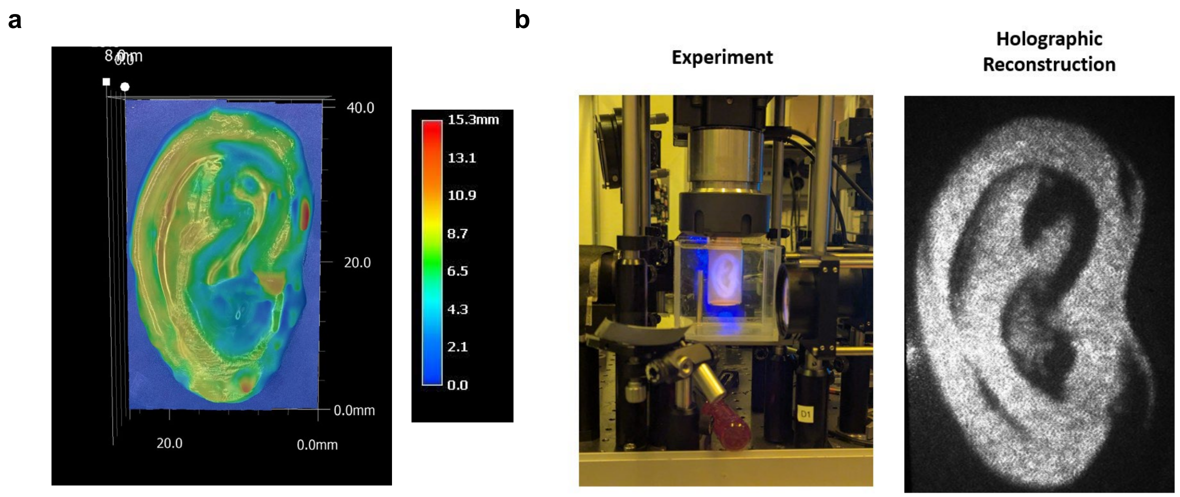

Fig S7.1. a. 3D stitching composition of example of a large-scale $(3 \times 3 \times 4\ cm^3)$ object in Gel-norbornene resin with holographic VAM using PLM. b. Left, image of the printing process of the human ear model. Right, holographic projection used to print the large-scale model using axicon shifts.

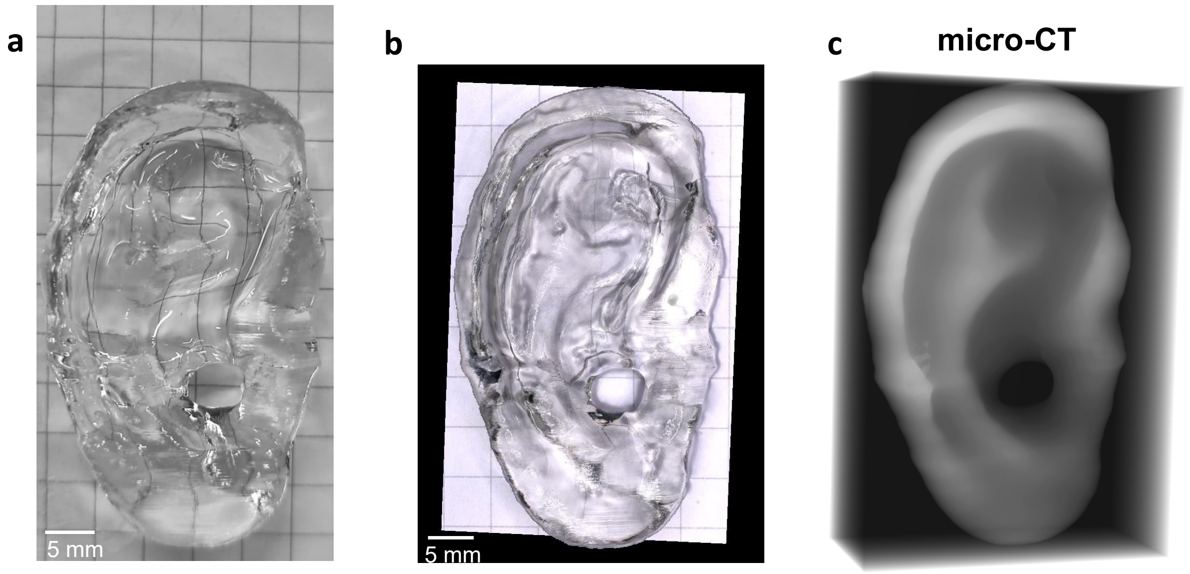

Fig S7.2. Example model of a large-scale human ear model printed in acrylate-based resin showing a smooth surface quality. a. Image of a large-scale human ear model printed with acrylate, placed over patterned paper to illustrate minimal surface scattering (transparency). **b**. 3D image composition profilometry of the large-scale Human ear model printed in acrylate. **c**. Micro-CT scan of the printed model, the scan resolution was $20\mu m$.

Supplementary Note 8: Post-processing measurements

To evaluate acrylate samples, we performed micro-CT scans and compared fidelity via representative feature measurements and Jaccard indices. Fig. S8 presents these measurements, including standard deviations, expected values, and model dimensions, illustrated through an example holographic projection. Fig. S8a (top and bottom) shows the corresponding phase pattern and intensity reconstruction simulation of the holographic projection at the given angle $\theta = 0°$, for both the small and large DNA helix models.

Measurements of the DNA cross-bars, which represent the smallest features printed with the technique, are reported in Fig. S8b for the small model and Fig. S8c for the large model. We report measurements of the lateral (XY) and cross-sectional (XZ) dimensions. As shown in the dashed area of Fig. S8a, the expected lateral size for the small model was $39.9\ \mu m$; however, the printed sample exhibits $38.13\ \mu m$. Similarly, the size of the printed cross-bars was measured to be $47.15\ \mu m$ instead of $53.2\ \mu m$.

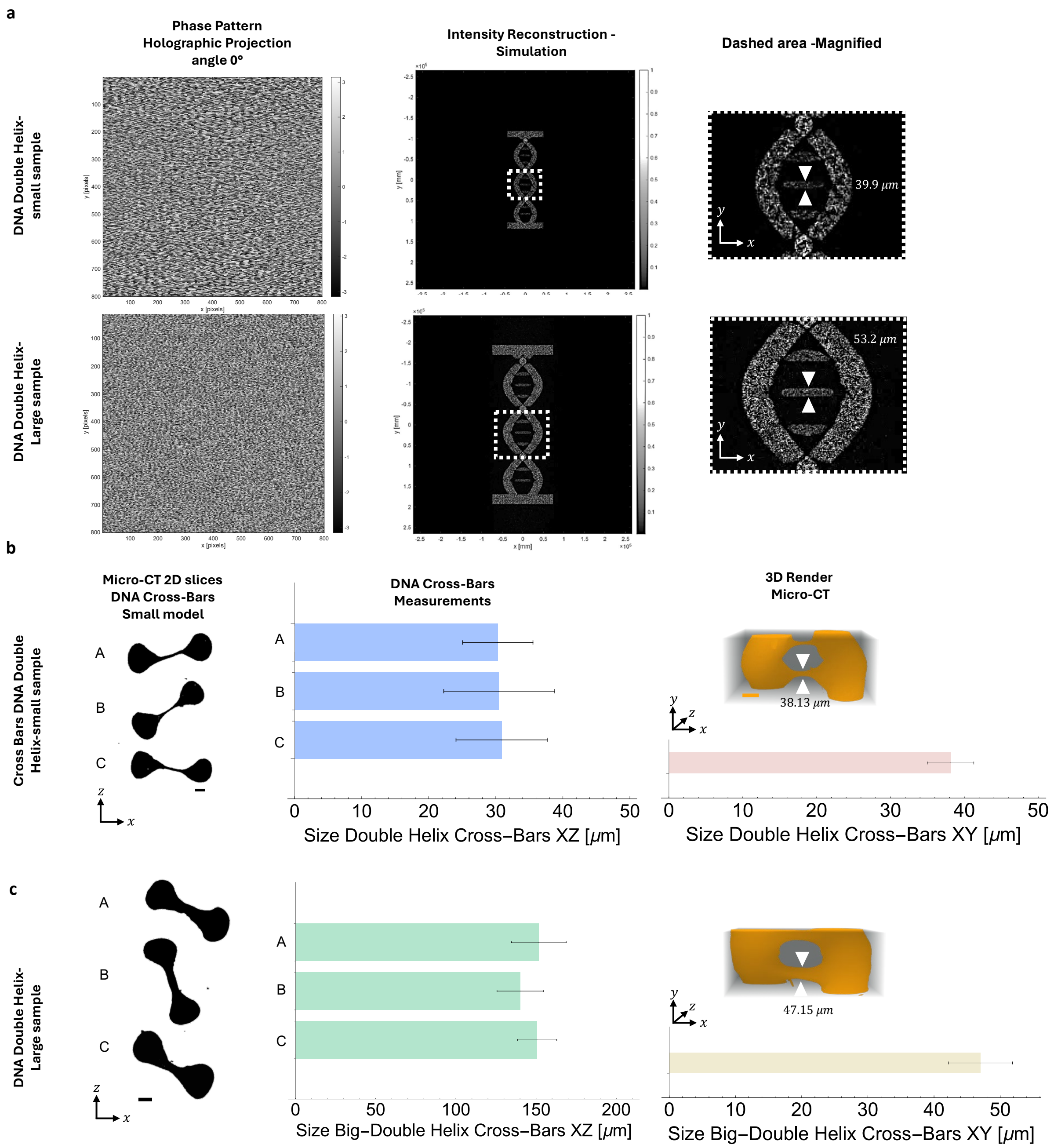

Fig. S8. DNA double helix projection and micro-CT scan analysis. a. Left: holographic projection (the phase pattern modulus 2π, prior to the encoding process required for the PLM, calculated using a phase retrieval algorithm). Center: simulation of the intensity reconstruction corresponding to the holographic projection. Right: magnified area corresponding to the double-helix cross-bars, showing the expected dimensions to be printed.

b. DNA helix small-model highlights. Left: 2D micro-CT slices through three cross-bars of the same sample (scale bars: $100\ \mu m$); Center: bar chart of the three cross-bar measurements from the printed object (error bars represent

standard deviations); Right: 3D micro-CT rendering of one cross-bar, with measurements illustrated in the bar chart (error bars represent standard deviations). **c**. DNA helix large-model highlights. Left: 2D micro-CT slices through three cross-bars of the same sample (scale bars: $200\ \mu m$); Center: bar chart of the three cross-bar measurements from the printed object (error bars represent standard deviations). Right: 3D micro-CT rendering of one cross-bar, with measurements illustrated in the bar chart (error bars represent standard deviations).

We calculated the Jaccard index to evaluate the fidelity of the printed samples. Overall, the Jaccard index for samples fabricated with the speckle noise reduction (stria control) technique indicates a fidelity of 0.83. The two large-model DNA helix samples printed with and without speckle reduction (stria control) show a difference of 0.3, demonstrating that stria control not only improves surface quality but also enhances overall printing fidelity. The small-model DNA helix was also printed using the stria control projection; however, its lower fidelity is attributed to diffusion effects associated with printed features smaller than 50 μm.

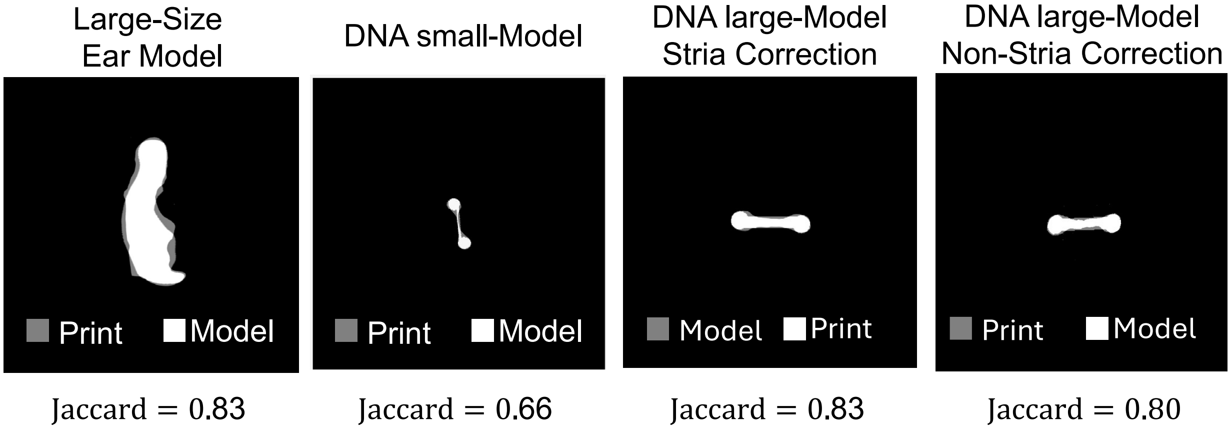

Fig S8.1. Jaccard index values for: Left, Human ear model. Center, Small-model DNA helix. Right, Larg-model DNA helix.

Supplementary References

[1] M. I. Álvarez-Castaño *et al.*, "Holographic tomographic volumetric additive manufacturing," *Nature Communications*, vol. 16, no. 1, p. 1551, Feb. 2025, doi: 10.1038/s41467-025-56852-4.

[2] D. Loterie, P. Delrot, and C. Moser, "High-resolution tomographic volumetric additive manufacturing," *Natature Communications*, vol. 11, no. 1, p. 852, Feb. 2020, doi: 10.1038/s41467-020-14630-4.

[3] W.-H. Lee, "COMPUTER-GENERATED HOLOGRAMS TECHNIQUES AND APPLICATIONS".

[4] Carmelo Rosales-Guzmán and Andrew Forbes, *How to Shape Light with Spatial Light Modulators*. SPIE Press, 2017.: https://doi.org/10.1117/3.2281295

[5] Z. Zhai *et al.*, "Parallel Bessel beam arrays generated by envelope phase holograms," *Optics and Lasers in Engineering*, vol. 161, p. 107348, Feb. 2023, doi: 10.1016/j.optlaseng.2022.107348.
